# Genetic Variation of Spongy Moth (*Lymantria dispar*) in Kazakhstan

**DOI:** 10.3390/insects17060591

**Published:** 2026-06-04

**Authors:** Alibek Makhambetov, Zarina Dairbekova, Bakyt Dulat, Abay Sagitov, Alexandr Pozharskiy, Yerlan Kydyrbayev, Allah Bakhsh, Dilyara Gritsenko

**Affiliations:** 1 Laboratory of Molecular Biology, Institute of Plant Biology and Biotechnology, Almaty 050040, Kazakhstan; alibekmahambetov@gmail.com (A.M.); dairbekovaz2001@gmail.com (Z.D.); bahytalt99@gmail.com (B.D.); a_sagitov@mail.ru (A.S.); aspozharsky@gmail.com (A.P.); 2 Department of Molecular Biology and Genetics, Al-Farabi Kazakh National University, Almaty 050040, Kazakhstan; 3 Research Center AgriBioTech, Almaty 050040, Kazakhstan; 4 Institute of Biology, National Academy of Sciences of the Kyrgyz Republic, Bishkek 720071, Kyrgyzstan; yerlankydyrbay@gmail.com; 5 Centre of Excellence in Molecular Biology, University of the Punjab, Lahore 54000, Pakistan; abthebest@gmail.com

**Keywords:** *Lymantria dispar*, genetic diversity, spongy moth

## Abstract

The spongy moth (*Lymantria dispar*) is a significant defoliator of broadleaf forests and fruit trees, but its population genetic structure in Central Asia remains poorly defined. We analyzed 153 specimens collected from ten populations across Kazakhstan between 2023 and 2024, using a combination of diagnostic real-time PCR, RAPD genotyping, and mitochondrial COI sequencing. Subspecies identification and Asian introgression were initially assessed with the COI/FS1 TaqMan assay. All samples carried the European mitochondrial type *L. dispar dispar*, while the nuclear FS1 locus indicated Asian introgression in 92.8% of specimens, including eleven heterozygotes for the Asian allele. RAPD amplification with five polymorphic primers produced 107 variable bands, which we converted into a presence/absence matrix for population genetics analysis. Principal coordinate analysis showed weak but detectable population structure, and Bayesian clustering (STRUCTURE) supported the presence of two primary nuclear clusters. Populations from Ketpentau, Sumbe, Kazachka, Butakovka, and Pavlodar were assigned predominantly to one cluster, whereas most individuals from Almaty, Ile-Alatau, Koksu, North Kazakhstan, and Tekeli were assigned predominantly to the other, with limited admixture in several localities. COI sequences formed a single, minimally differentiated *L. dispar* clade, with no evidence of distinct mitochondrial lineages. Overall, these findings reveal extensive Asian nuclear introgression in populations retaining European mitochondrial haplotypes, supporting the interpretation of Kazakhstan as part of a broad contact zone within the larger *dispar–asiatica* gradient. The observed mito-nuclear discordance has important implications for quarantine diagnostics and highlights the need for multilocus molecular assays in regional biosurveillance.

## Simple Summary

The spongy moth (*Lymantria dispar*) is a quarantine forest pest that regularly causes defoliation of broadleaf trees and threatens both natural ecosystems and fruit production. In Kazakhstan, outbreaks have been recorded for decades, but almost nothing was known about the genetic composition of local populations. In this study, we combined modern molecular tools to analyze 153 specimens collected from ten localities across northern and southern Kazakhstan. All moths carried a European mitochondrial haplotype, yet over 90% also possessed nuclear markers typical of the Asian subspecies, indicating extensive hybridization between the two lineages. Population-genetic analyses based on RAPD markers revealed two main genetic groups arranged along a broad north–south axis, with strongly admixed populations in large urban and peri-urban areas such as Almaty. These patterns suggest that Kazakhstan acts as a hybrid contact zone where natural dispersal and human-mediated transport promote mixing of European and Asian gene pools. Our results show that apparently “European” populations may still harbor high-risk Asian alleles, underscoring the need to incorporate molecular diagnostics into national monitoring and management programs for this pest.

## 1. Introduction

The spongy moth, previously known as the gypsy moth (*Lymantria dispar* Linnaeus (Lepidoptera: Erebidae)), is a highly destructive forest pest widely distributed across Europe, Asia, North Africa, and North America [1]. Its life cycle consists of four stages: egg, larva, pupa, and adult. The larval stage is particularly hazardous, as larvae can cause extensive defoliation, destroying entire forests. Depending on the subspecies, these larvae can consume foliage from approximately 300 tree species, making them among the most versatile and dangerous defoliators [1].

According to the International Union for Conservation of Nature, the spongy moth is among the 100 worst invasive species globally [1]. Females may lay 200 to 1200 eggs on tree branches and trunks, which can lead to complete defoliation of trees and shrubs, making them more vulnerable to infection [1,2].

The ability of spongy moths to cause extensive defoliation across a wide variety of tree species results in severe ecological and economic damage, necessitating ongoing research and control measures [1,2]. Three subspecies of *L. dispar* are recognized as the most significant and extensively studied: the European spongy moth (*Lymantria dispar dispar* Linnaeus), the Asian spongy moth (*Lymantria dispar asiatica* Vnukovskij), and the Japanese spongy moth (*Lymantria dispar japonica* Motschulsky) [3].

*Lymantria dispar dispar* is native to the Palearctic region and is widely distributed across much of Europe and parts of North Africa and western Asia, where it is particularly abundant in oak-dominated temperate forests of Central and Southern Europe, the Near East, and North Africa, and is regarded as a major defoliating forest pest [1,4,5]. In 1869, Étienne Léopold Trouvelot intentionally imported *L. dispar dispar* from France into Medford, Massachusetts, to develop a stronger silkworm hybrid. However, some larvae escaped and formed feral populations [6,7,8]. Since then, the European spongy moth has expanded across the northeastern United States into southeastern Canada and is now regarded as one of the most damaging invasive forest insects in North America [1,7,8,9].

*Lymantria dispar asiatica* Vnukovskij is widely distributed across Asia, ranging from the Ural Mountains and Central Asian countries in the west to the Russian Far East, including Mongolia, the northern two-thirds of China, and the Korean Peninsula [10]. Some authors distinguish four geographical variants for Asian spongy moths based on ecological and behavioral characteristics: Central Asian, Western Siberian, Eastern Siberian, and Far Eastern variants [10,11,12,13]. The Central Asian variant has been found in southern Kazakhstan, Uzbekistan, Kyrgyzstan, Tajikistan, Turkmenistan, northern Afghanistan, and the Xinjiang Autonomous Region of China. The Western Siberian variant is local to the southwestern part of Asiatic Russia and northern Kazakhstan. The Eastern Siberian variant occupies territory from south-central Siberia to the foothills of the Stanovoy Range in the east, as well as Mongolia and parts of China. The Far Eastern variant includes populations in northeastern China, the Korean Peninsula, and the southern Russian Far East [10,11,12,13].

*Lymantria dispar japonica* Motschulsky is native to Japan. This subspecies is distributed across all four major Japanese islands: Honshu, Shikoku, Kyushu, and parts of southern and western Hokkaido. It inhabits various forested regions, where it plays a role in the local ecosystem [10,14].

All three subspecies are polyphagous [1,15,16], but the last two exhibit a broader host range compared to *L. dispar dispar* [1,17,18]. Furthermore, Asian subspecies females can sustain long flights, whereas European subspecies females are flightless [1,2,19]. Egg-laying behavior in the Asian spongy moth is highly influenced by light conditions. Additionally, the temperature needed for hatching varies notably between subspecies, with *L. dispar asiatica* eggs needing less exposure to cold temperatures to hatch than *L. dispar dispar* eggs [2].

Insufficient knowledge of genetic diversity in quarantine pests can hinder accurate subspecies identification, mask introgression between lineages, and complicate the reconstruction of invasion pathways, thereby reducing the effectiveness of biosurveillance and management programs. Recent studies of Asian spongy moth populations in China have revealed new haplotypes, underestimated mitochondrial diversity, and unexpectedly high genetic diversity, indicating that earlier diagnostic approaches based on limited reference material may not be sufficiently robust for all regional populations and supporting the need for improved molecular identification methods [2,3,20,21].

Asian introgression in the spongy moth involves hybridization and gene flow from the Asian subspecies—*L. dispar asiatica* and *L. dispar japonica*—into populations of the flight-restricted European subspecies, *L. dispar*. Range-wide nuclear genetic and genomic studies have shown that the genetic structure of *Lymantria dispar* across Eurasia does not fully correspond to the traditionally recognized subspecies boundaries, but instead indicates a broad transition from Asian-associated populations in East Asia toward European-associated populations in Western Europe, with evidence of admixture in intermediate regions, including Central Asia [22,23]. The biological importance of this introgression encompasses several adaptive traits inherited from the Asian lineage, particularly fully developed, flight-capable females, increased host range, and phenological plasticity. Comparative flight experiments showed that Asian females were able to sustain directed flights of 1–2 km, while European females were essentially unable to fly [1,24]. These traits enhance the natural dispersal ability and increase the risk of invasion whenever Asian alleles are integrated into European backgrounds.

Evidence of Asian introgression has now been detected well beyond the native contact zone. A multiplex real-time PCR assay targeting the diagnostic FS1 allele has identified Asian haplotypes in spongy moth specimens intercepted at North American ports, suggesting multiple introductions and occasional hybridization events with existing European populations [7]. Population-genomic reconstructions further indicate that multiple North American colonies contain low-frequency Asian nuclear segments, confirming that introgression can persist after initial establishment [2,24]. Since introgressed females might regain some flight capability, even infrequent hybridization events raise regulatory issues: models that account for female flight suggest quicker range expansion and diminished effectiveness of pheromone-based containment methods [1].

This genetic mixing can obscure traditional morphological or molecular markers used to differentiate species, making accurate identification more challenging and underscoring the need for updated, sophisticated genetic analysis techniques to monitor and manage these populations effectively [24].

Despite extensive research, *L. dispar* remains a quarantine pest in several countries worldwide due to its high invasiveness and destructive impact on forest ecosystems, particularly when eradication measures fail. In the United States, *L. dispar* has been under quarantine status since 1989, while in Europe it has been listed in the A1 category by the European and Mediterranean Plant Protection Organization (EPPO) for countries such as Azerbaijan (since 2007) and Georgia (since 2018) [1,25]. In the Russian Federation, *L. dispar* (including the Asian form *L. dispar asiatica*) has been listed as an A2 quarantine pest under the EPPO framework since 2014, although the Asian form has been regulated as an A2 quarantine pest in Russian national legislation since 1992 [25,26]. With regard to Central Asia, the Asian subspecies *L. dispar asiatica* has been regulated as a quarantine pest of limited distribution in the Republic of Kazakhstan since at least 2015, when it was included in the national list of quarantine plant pests. This status was reaffirmed in the EPPO A2 list for Kazakhstan in 2017, coinciding with repeated outbreaks and range expansion, particularly in the country’s southern and southeastern regions [1,27,28].

However, despite the broad distribution and quarantine importance of *L. dispar*, the genetic structure of its populations in Kazakhstan remains insufficiently studied. This gap is particularly important because Kazakhstan lies in a geographically intermediate part of Central Asia, where contact between European- and Asian-associated genetic lineages of *Lymantria dispar* may occur.

Mitochondrial COI has been widely used for species identification, DNA barcoding, and phylogeographic analysis of *L. dispar*, including studies from Europe, East Asia, and North America [2,3,7]. However, available COI data from Kazakhstan are very limited, due to previous studies having included only a limited number of specimens from Kazakhstan or neighboring regions, and these data were usually analyzed within broader range-wide datasets rather than as a focused population-level assessment of Kazakhstan populations [1,2,6,29]. Moreover, COI reflects only the maternally inherited mitochondrial lineage and therefore cannot fully describe nuclear ancestry or detect introgression in potential contact zones [1,6,29]. This limitation is particularly relevant for Central Asia, where earlier work indicated that specimens from Kazakhstan and Kyrgyzstan may possess mixed European and Asian genetic backgrounds [1,29].

At the nuclear-genome level, previous microsatellite and SNP-based studies have shown that Central Asian populations, including material from Kazakhstan and Kyrgyzstan, may represent a zone of admixture between European and Asian genetic backgrounds [6,9,29]. Nevertheless, Kazakhstan has not yet been examined in detail using a dense population-level sampling scheme across multiple localities. Therefore, the contribution of the present study is to provide a focused assessment of *L. dispar* populations in Kazakhstan by combining COI sequencing, COI/FS1 TaqMan assays, and multilocus RAPD profiling. This combined approach allows us to evaluate whether mitochondrial and nuclear markers reveal concordant or contrasting patterns, and to clarify the role of Kazakhstan as a potentially underexplored contact zone between European and Asian spongy moth lineages. The present study aims to fill this gap by providing the first molecular data for large populations of the spongy moth in Kazakhstan. The results will clarify the pest’s invasion and distribution and thus assist in the conservation of the region’s wild forests and gardens.

## 2. Materials and Methods

### 2.1. Collection of Spongy Moth Samples and DNA Extraction

**Figure 1:**
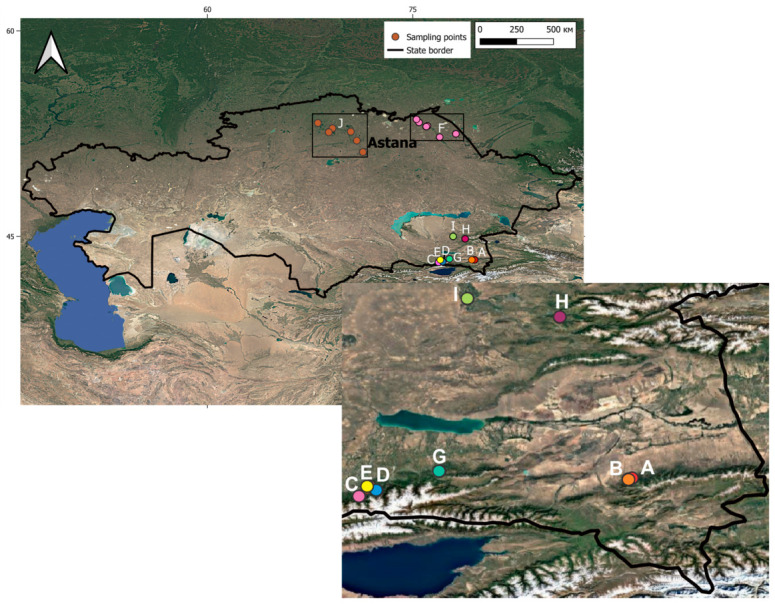
Sampling locations. Colored circles indicate sampling points, and letters indicate the population codes. The main map shows the distribution of sampling sites across Kazakhstan, while the enlarged inset provides a detailed view of the southeastern sampling area, where most populations were collected. The rectangular boxes are used to visually highlight areas with closely located sampling points.

A total of 153 spongy moth samples were collected during the summer of 2023 and 2024 from different host plants, including rosehip (*Rosa* spp.), apple (*Malus* spp.), barberry (*Berberis* spp.), hawthorn (*Crataegus* spp.), and oak (*Quercus* spp.) (Figure 1, Table 1 and Table S1). A total of 133 larvae samples were collected manually, using visual search, from eight locations in Kazakhstan—Ketpentau, Sumbe, Kazachka, Butakovka, Almaty, Pavlodar, Ile-Alatau, and Tekeli—and 20 adult male specimens were collected from Koksu and North Kazakhstan using delta pheromone traps with commercial lures (rubber dispenser containing the female sex pheromone of *L. dispar*). The lure was placed on the adhesive insert inside the delta trap, and traps were deployed in forested sampling sites during the adult flight period. Some late-instar larvae collected shortly before pupation were maintained under controlled laboratory conditions at 24 °C and 64% relative humidity, where, after pupation, the adult male and female specimens emerged. The collected specimens of spongy moth larvae and adult were examined visually, photographed and preserved in 96% ethanol and stored at +4 °C prior to DNA extraction.

Genomic DNA was extracted from both larval and adult specimens of the spongy moth using the protocol described by Marín D.V. et al. [30] with minor modifications. In adult moths, DNA was extracted from the head and thorax, while in larval specimens the head and thoracic segments were used as the tissue source. Ethanol-preserved samples were cut using sterile scalpels and air-dried for approximately 30 min before being homogenized using liquid nitrogen. Lysis was performed for 30 min at 65 °C in 400 µL CTAB buffer (4% CTAB, 100 mM Tris-HCl pH 8.0, 1.4 M NaCl, 25 mM EDTA, 0.4% β-mercaptoethanol, 2% polyvinylpyrrolidone). Two sequential extractions were performed: first with chloroform:isoamyl alcohol (24:1), then with pure chloroform, to remove proteins and other contaminants. Each step was followed by centrifugation at 16,500× *g* for 6 min. The clear supernatant was carefully transferred to fresh 1.5 mL tubes and precipitated overnight at −20 °C with an equal volume of cold isopropanol. Following centrifugation at 16,000× *g* for 12 min, the DNA pellet was washed with 90% ethanol, then with 70% ethanol, air-dried, and finally resuspended in 50 µL of nuclease-free water. Residual RNA was digested with RNase A (37 °C, 1 h). DNA purity and concentration were measured using a NanoDrop One spectrophotometer (Thermo Fisher Scientific, Waltham, MA, USA). The extracted DNA concentration was adjusted to 50 ng/µL for PCR amplification.

### 2.2. Real-Time PCR

For subspecies-level diagnostics, the primer/probe panels developed by Stewart et al. [24] were used. The mitochondrial COI diagnostic assays included codes 1A, 1B, 2B, and 3A, which were applied for the identification of *L. dispar* subspecies and related Asian spongy moth diagnostic groups. In particular, assay 3A was used to detect the *L. d. dispar* mitochondrial type, whereas assays 1A/1B and 2B were used to screen for Asian and Japanese spongy moth-associated COI diagnostic profiles. The nuclear FS1 assay 4A/4B was used to evaluate the presence of Asian-associated nuclear introgression in specimens identified as *L. d. dispar* by mitochondrial markers. Primer and hydrolysis probe sequences, as well as the diagnostic scheme, were taken from the original publication. Reactions were set upon a total volume of 20 µL, comprising 10 µL of 1× Luna Universal Probe qPCR Master Mix (New England Biolabs, Ipswich, MA, USA), 1 µL each of forward and reverse primers, 0.5 µL of probe, 2 µL of DNA template (100 ng), and nuclease-free water to volume. Based on the published genome size of *L. dispar* (≈998 Mb for *L. d. dispar* and 921–999 Mb for Asian subspecies) and the estimated genome mass (≈1.03 pg per haploid genome), 1 µg of *L. dispar* genomic DNA corresponds to approximately 9.7 × 10^5^ genome copies, i.e., one copy of the haploid genome corresponds to ~1.03 pg (1030 fg) of DNA. At a working concentration of ~50 ng DNA per reaction, approximately 4.9 × 10^4^ genome copies were added per well, providing reliable detection of specific TaqMan signals for all markers used. Thermal profile: initial denaturation at 95 °C for 15 min; then 40 cycles of 95 °C for 15 s and 50 °C for 30 s; final extension at 65 °C for 1 min. The real-time PCR was performed on a CFX Opus 96 real-time PCR detection system (Bio-Rad Laboratories Inc., Hercules, CA, USA) with the fluorescence signal recorded at each cycle, at the end of the extension step; the lid temperature was maintained at 105 °C, and data processing was performed in CFX Manager v3.1 (Bio-Rad Laboratories Inc., Hercules, CA, USA). The results of the assay were interpreted according to the protocol described in the original publication.

### 2.3. Genetic Diversity

#### 2.3.1. PCR with RAPD Markers

To assess the genetic diversity of *L. dispar* populations, we employed RAPD-PCR, which uses short, arbitrary 10 bp primers. Five universal primers—OPB-04, OPN-09, OPY-15, OPAN-03, and UBC378 [31,32,33,34,35,36,37,38,39]—were chosen after confirming that their sequences have multiple complementary regions in the reference genome assembly of *L. dispar* (GCA_032191425.1 [40]). In silico RAPD analysis was performed using a custom pipeline. Primer binding sites were identified using EMBOSS fuzznuc, allowing up to two mismatches [41]. Hits were filtered based on perfect matching of the last four 3′ nucleotides. Forward and reverse sites were paired to generate potential amplicons (100–3000 bp). Amplicons were classified based on mismatch levels at both primer binding sites, and their distribution across chromosomes was visualized using custom Python (3.14.3) scripts that used the Pandas (3.0.2) and Matplotlib (3.10.8) libraries [42,43].

The PCR reaction mixture (25 μL) for every RAPD primer contained 1 μL of template DNA (50 ng/µL), 1 µL of primer (10 µM), 0.5 µL of dNTP (10 mM), 2.5 μL of 1× Standard Taq Reaction Buffer (New England Biolabs, Hercules, CA, USA) and 0.2 μL of Taq DNA Polymerase (New England Biolabs, Hercules, CA, USA). Using a C1000 Touch Thermal Cycler (Bio-Rad Laboratories, Hercules, CA, USA), PCR was run with a preheating step at 94 °C for 5 min followed by 40 cycles of denaturation at 94 °C for 45 s, annealing at 34.5 °C for 45 s, polymerization at 72 °C for 80 s and final extension at 72 °C for 10 min. PCR products were visualized by electrophoresis on 1,5% agarose gel, with 1× TAE buffer (40 mM Tris, 20 mM acetate, and 1 mM EDTA). Each distinct band was considered a RAPD marker; the frequency of each marker was estimated for each population. The sizes of the resulting fragments were determined by comparison with a molecular size marker (1 Kb Plus DNA Ladder by TermoFisher Scientific, Waltham, MA, USA and GeneRuler 1 Kb Plus DNA Ladder by Thermo Scientific, Waltham, MA, USA). In total, ten populations were established based on the corresponding sampling localities. The data on the RAPD markers have been coded as a binary matrix with “1” corresponding to the presence and “0” to the absence of the band.

To validate the RAPD profiles and ensure the reproducibility of the analysis, PCR amplification was performed in three independent replicates for each sample and primer combination. Only clear, well-resolved bands that were consistently present in all three replicates were scored and included in the binary matrix. Weak, ambiguous, or non-reproducible bands were excluded from further analysis.

The population structure was analyzed using STRUCTURE 2.3.4 [44] under an admixture model with correlated allele frequencies and prior population information. The analysis was performed with a burn-in period of 50,000 iterations followed by 100,000 MCMC iterations. The number of clusters (K) ranged from 1 to 10, with 10 independent runs per K. The results were visualized and summarized using the CLUMPAK (1.1) [45], and the optimal K value was determined according to Evanno’s method [46].

Genetic relationships among individuals were further explored using a distance-based framework based on RAPD presence/absence data. A Jaccard dissimilarity matrix was calculated in R (4.5.3) using the vegdist function from the vegan package (method = “jaccard”, binary = TRUE) [47,48]. Principal coordinate analysis (PCoA) was performed using classical multidimensional scaling (cmdscale) to visualize patterns of genetic variation [49].

Genetic differentiation among populations was quantified using a distance-based, permutation-based analysis of molecular variance (AMOVA-like) implemented in the R environment (version 4.5.2) with the vegan package [47,48,50]. A one-factor model was applied to partition genetic variation into components attributable to differences among populations and within populations. Statistical significance was assessed using 999 permutations.

The proportion of variation explained by population grouping (R^2^) was used as an analog of PhiPT (ΦPT), representing genetic differentiation for binary RAPD data and conceptually analogous to Fst [49,51]. Pairwise genetic differentiation between populations was further evaluated using the same permutation-based framework applied to pairwise subsets of the distance matrix.

#### 2.3.2. COI Sequencing

To further assess the genetic diversity of the populations, high-throughput Nanopore sequencing of the COI region amplicon was performed. The amplification of the COI (cytochrome oxidase I) gene was performed using the LCO1490 and HCO2198 primers described by Folmer et al. (1994) [52]. The PCR reaction mixture (25 μL) contained 1 μL of template DNA (50 ng/µL), 1 µL of primer (10 µM), 0.5 µL of dNTP (10 mM), 2.5 μL of 1× Standard Taq Reaction Buffer (New England Biolabs, Ipswich, MA, USA) and Taq DNA Polymerase (New England Biolabs, Hercules, CA, USA). The amplification program on C1000 Touch Thermal Cycler (Bio-Rad Laboratories, Hercules, CA, USA) included an initial denaturation at 95 °C for 2 min, followed by 5 preliminary cycles, each consisting of denaturation at 95 °C for 1 min, primer annealing at a reduced temperature of 46 °C for 1 min, and elongation at 72 °C for 30 s. Subsequently, 34 main amplification cycles were performed using the same scheme, except with an increased annealing temperature of 53 °C. A final extension step was carried out at 72 °C for 5 min after the completion of all cycles.

The obtained PCR products were stored at 4 °C until further use. Analysis of the PCR products was performed by horizontal electrophoresis in a 1.5% agarose gel. Library preparation was conducted using a Rapid Barcoding Kit (SQK-RBK114.96; Oxford Nanopore Technologies, Oxford, UK) on PromethION P2 Solo sequencing device (Oxford Nanopore Technologies, Oxford, UK); following the manufacturer’s protocol, 1.5 µL of unique barcodes were added to all the samples. Then all samples were pooled and cleaned using AmPure XP beads (Beckman Coulter, Brea, CA, USA), provided in the kit, followed by washing with freshly prepared 80% ethanol. The following step included adding the Rapid Adapter to regulate the DNA passage speed through nanopore. Subsequently, the priming mix and library mix were loaded onto the FLO-PRO114M flow cell. Sequencing was managed using MinKNOW software (v25.05.14), with the launch settings of a minimal PHRED score of 9. The generated raw data was basecalled using Dorado (v7.9.8) with a high accuracy basecalling model. Initial quality control was conducted using NanoFilt (2.8.0) [53] to extract reads with q > 12 and a length > 200 bp. A reference for the COI gene was assembled from the NCBI database. Mapping was performed using minimap2, and the consensus was generated using samtools (1.23.1). The resulting sequences were aligned in UGENE software (53.1) [54] using the MAFFT (7.526) method [55]. The phylogenetic tree was assembled in MEGA12 using the Maximum Likelihood model and Tamura–Nei substitution model [56]. 

## 3. Results

### 3.1. Morphological Identification of Spongy Moth Subspecies

**Figure 2:**
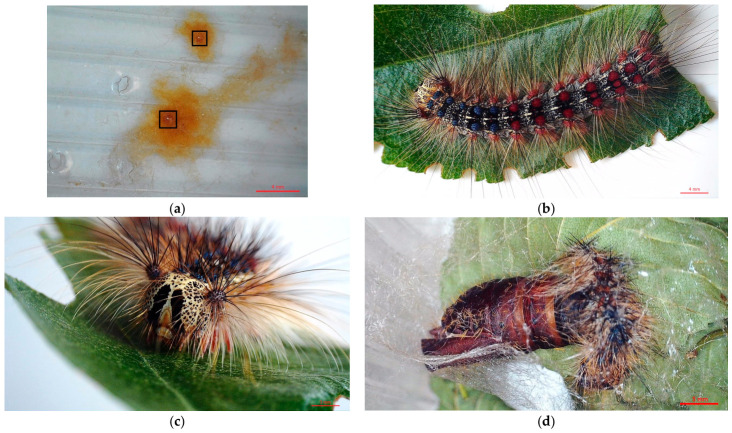

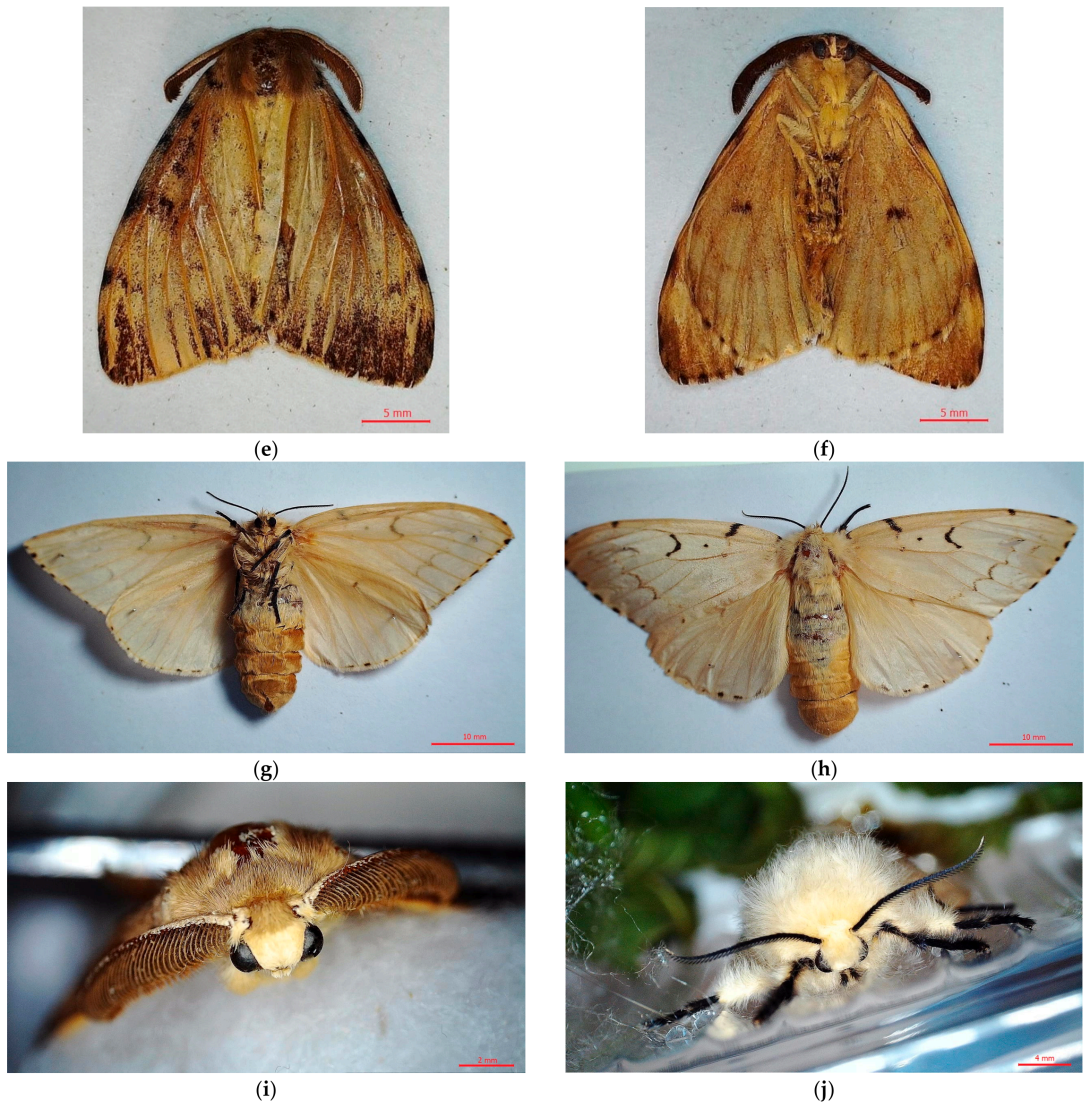
*L. dispar* specimens at different life stages: (**a**) Eggs outlined with black rectangles; (**b**,**c**) Larvae; (**d**) Pupa; (**e**,**f**) Adult male specimen dorsal and ventral view; (**g**,**h**) Adult female specimen ventral and dorsal view; (**i**) Adult male specimen frontal view; (**j**) Adult female specimen frontal view.

The samples showed typical morphological features of *L. dispar* (Figure 2). Egg masses were typically deposited as compact, oval to teardrop-shaped plaques on bark, branches, stones, or other sheltered surfaces. The egg mass is light brown and covered by yellowish-brown abdominal hairs from the female, giving it a spongy appearance (Figure 2a). Individual eggs are approximately 1 mm in diameter and lay hidden beneath the hair layer.

Larvae were mottled yellow-gray with prominent verrucae bearing tufts of bristle-like hairs, as described for spongy moths in standard keys [4,6,25]. Mature larvae (final instar) reached approximately 4–6 cm in length and exhibited the characteristic dorsomedian pattern of five pairs of blue tubercles followed by six pairs of red tubercles on the first eleven abdominal segments, a diagnostic feature that reliably separates *L. dispar* from other sympatric defoliators [24] (Figure 2b). The head capsule of older larvae was predominantly yellow to yellow-brown with darker mottling, again matching reference descriptions for *L. dispar* larvae [57] (Figure 2c). No specimens with deviant tubercle patterning or coloration suggestive of other *Lymantriine* species were observed.

Pupae of *Lymantria dispar* had a fusiform to teardrop-shaped body, dark reddish-brown to dark brown coloration, and the presence of sparse yellowish to reddish setae on the body surface. The pupae were attached to the substrate by loose silken threads, usually in protected sites such as bark crevices, under branches, or other sheltered surfaces. Female pupae measured approximately 15–35 mm, while male pupae were shorter, at approximately 15–20 mm (Figure 2d).

Adult males obtained from the same populations were also morphologically consistent with *L. dispar* (Figure 2e,f,j). Males had a slender, dark-brown body and strongly bipectinate antennae, with a wingspan of approximately 30–40 mm. Forewings were tan to gray-brown with irregular darker wavy bands and blackish markings, including a reniform spot and a series of small terminal dots, while hindwings were paler yellowish to brown [25,58].

Adult females are larger and more robust than males, with a stout abdomen covered with pale hairs and a wingspan of approximately 55–70 mm (Figure 2g–i). The wings are predominantly white to grayish-white and marked with dark, saw-toothed or wavy transverse patterns. Females also possess pectinate antennae, but the branches are shorter than in males, making the antennae less feather-like.

### 3.2. Real-Time PCR Results

All 153 samples were successfully genotyped using the panel of specific TaqMan markers. The 18S rRNA internal control is consistently positive in all reactions, confirming the absence of PCR inhibition. The mitochondrial markers 1AB and 3A showed that none of the samples belonged to the Asian subspecies (no 1AB signal was detected in any sample), whereas all individuals carried the European mitotype detected by marker 3A. The nuclear marker FS1 (4AB), used to diagnose Asian introgression, revealed the presence of the Asian allele in 142 of 153 (92.8%) individuals examined: 137 specimens were homozygous and five were heterozygous for the Asian allele; the remaining 11 specimens carried only the European allele and showed no evidence of introgression. Thus, within a morphologically homogeneous sample of the European subspecies, molecular analysis revealed a high prevalence of Asian nuclear introgression in the surveyed populations.

In all positive samples, exponential growth of the fluorescent signals for markers 1AB, 3A, FS1, and 18S was observed before cycle 35; reactions in which signal increase occurred after 35 cycles were excluded from the interpretation of results after three repetitions. Samples with ambiguous amplification profiles were reanalyzed three times to confirm their status. In negative control reactions without template DNA (1 µL nuclease-free water), no specific signal was detected for any marker, indicating the absence of contamination and nonspecific amplification.

### 3.3. Genetic Diversity

#### 3.3.1. PCR with RAPD Markers Results

Genetic diversity in spongy moth populations was assessed using RAPD profiling, which detects polymorphism by PCR fragments using short (10-nt) random primers. Prior to experimental validation, the selected primers were screened in silico against the reference genome assembly of the spongy moth (*L. dispar*) to confirm the presence of multiple complementary binding sites and their potential to yield informative amplification profiles; this screening suggested that each primer would produce a characteristic set of amplicons, as shown in Table 2.

This approach allowed each clearly resolved band to be treated as an individual RAPD locus and the resulting profiles to be encoded as a binary matrix (1 = presence, 0 = absence).

**Figure 3:**
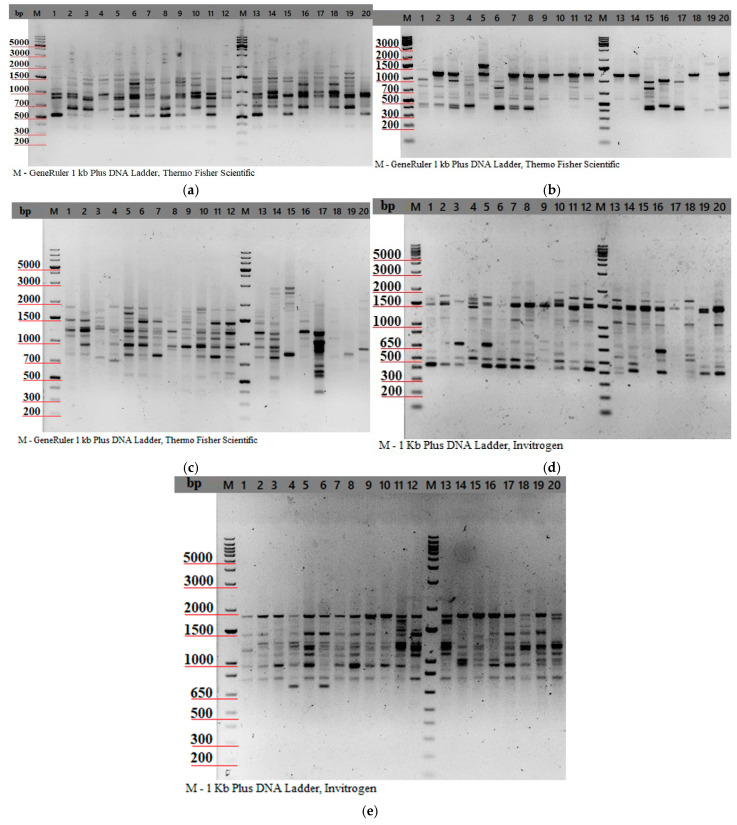
Representative RAPD-PCR amplification profiles of *Lymantria dispar* specimens from Kazakhstan. Agarose gel electrophoresis showing RAPD-PCR banding patterns generated with the five selected primers: (**a**) [OPAN-03], (**b**) [OPB-04], (**c**) [UBC378], (**d**) [OPY-15], and (**e**) [OPN-09]. Numbered lanes correspond to individual specimens, and M indicates the DNA ladder.

All five primers (OPB04, OPN-09, OPY-15, OPAN-03 and UBC378) produced clear and reproducible banding patterns and were retained for subsequent analyses (Figure 3 and Table S2). The PCR amplification with primers resulted in a total of 107 RAPD fragments, all of which varied among at least two populations and were classified as polymorphic. Specifically, OPB-04 produced 21 fragments of 410–1900 bp, OPN-09 produced 19 fragments of 700–2125 bp, OPY-15 yielded 21 fragments of 440–1800 bp, OPAN-09 generated 20 fragments of 300–2000 bp, and UBC378 produced 26 fragments of 450–2200 bp. Across all primers, fragment sizes ranged from 300 to 2200 bp, demonstrating the substantial resolving capacity of the selected RAPD marker system for evaluating intraspecific genetic variation in *L. dispar*.

**Figure 4:**
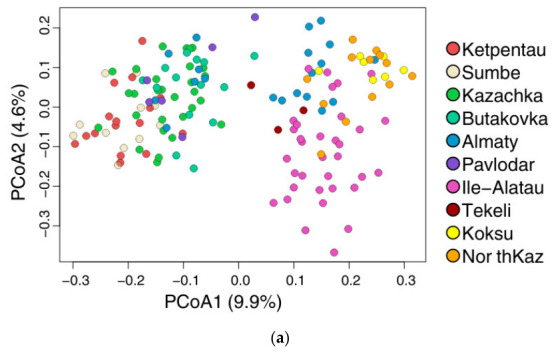

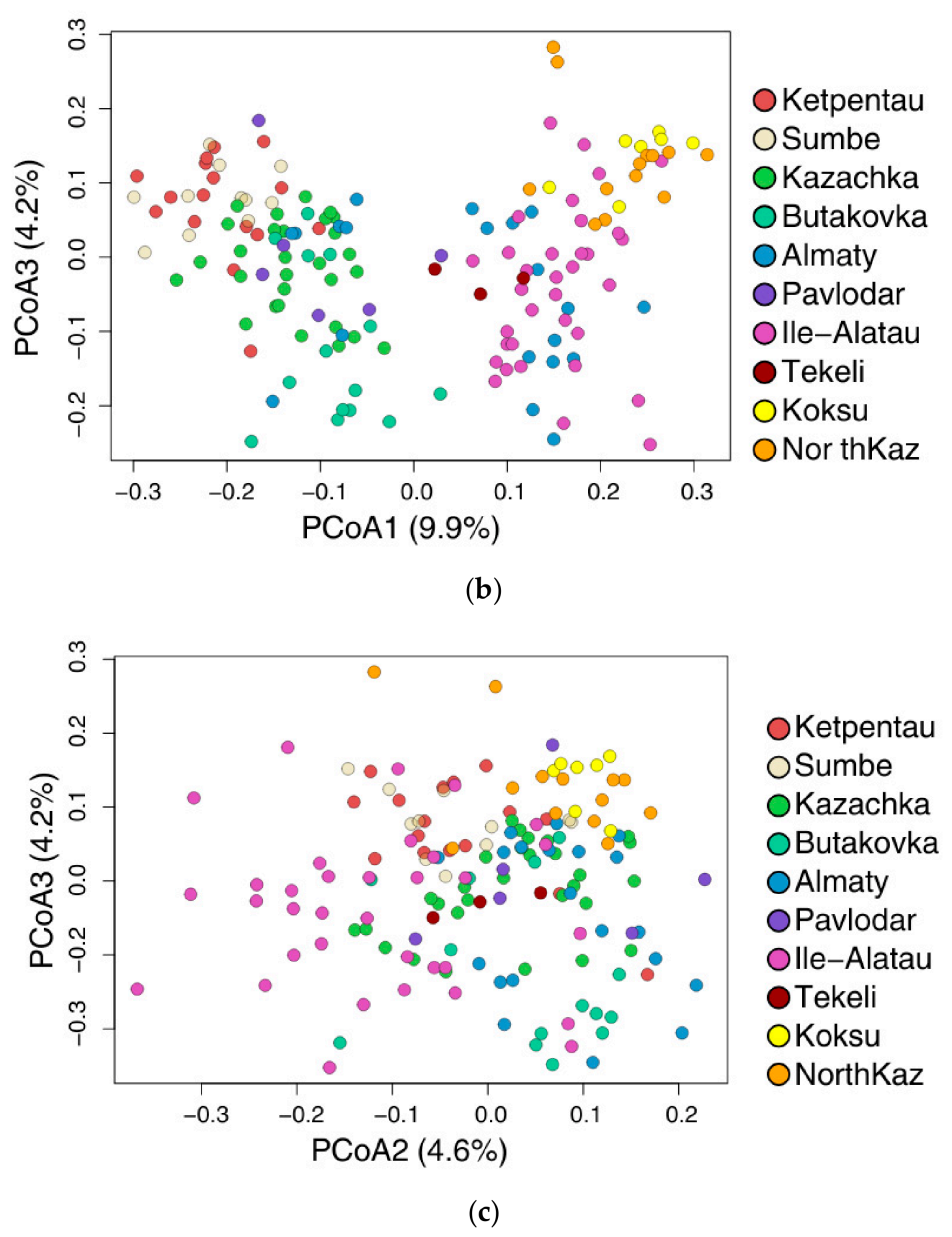
PCoA graph in two orientations. points are colored according to population of origin—Ketpentau (A), Sumbe (B), Kazachka (C), Butakovka (D), Almaty (E), Pavlodar (F), Ile-Alatau (G), Tekeli (H), Koksu (I), and North Kazakhstan (J)—corresponding to the population codes used in Figure 1 and Table 1. (**a**) Ordination of individuals along PCoA1 and PCoA2, explaining 9.9% and 4.6% of the total variation, respectively; (**b**) ordination of individuals along PCoA1 and PCoA3, explaining 9.9% and 4.2% of the total variation, respectively; (**c**) ordination of individuals along PCoA2 and PCoA3, explaining 4.6% and 4.2% of the total variation, respectively.

For subsequent analyses of genetic diversity and population structure, the presence or absence of each fragment of a given size was scored in a binary matrix (1/0). Principal Coordinate Analysis (PCoA) based on binary distances showed that the first three principal coordinates explained approximately 9.9%, 4.9%, and 4.3% of the total variance, respectively (Figure 4).

In the PCoA1–PCoA2 plane, individuals formed two broad and partially overlapping assemblages separated mainly along PCoA1. Ketpentau, Sumbe, Kazachka, and Butakovka were located predominantly on the negative side of PCoA1, whereas Ile-Alatau, Koksu, North Kazakhstan, and part of Almaty were shifted toward positive PCoA1 values. PCoA2 provided only limited additional separation, with Koksu and North Kazakhstan tending toward higher PCoA2 values and Ile-Alatau showing broader dispersion toward lower PCoA2 values. Importantly, the distribution of these two RAPD-based assemblages was not strictly consistent with geographic proximity. In the southern part of the sampling range, the geographically close populations Kazachka (C), Butakovka (D), Almaty (E), and Ile-Alatau (G) were divided between the two assemblages: Kazachka and Butakovka were mainly associated with the negative PCoA1 group, whereas Almaty and Ile-Alatau were shifted toward the positive PCoA1 group. A similar pattern was observed among the northern populations, where Pavlodar (F) was associated mainly with the first assemblage, while North Kazakhstan (J) was grouped with the second. Thus, the RAPD-based structure indicates two broad nuclear groups, but not a simple latitudinal or distance-based pattern.

**Figure 5:**
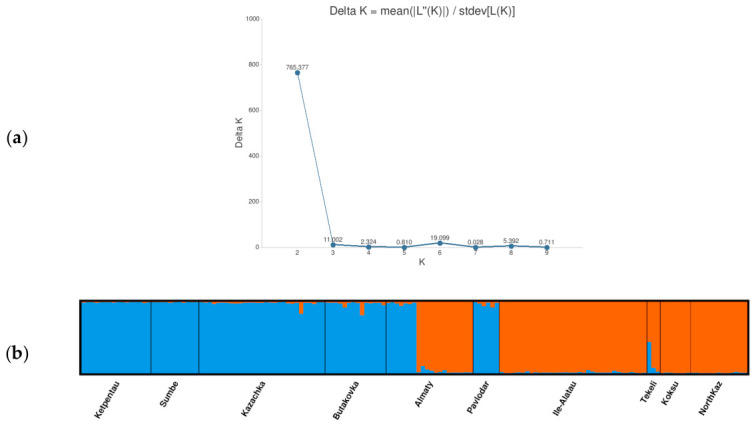
Bayesian clustering analysis in STRUCTURE: (**a**) Estimation of the most likely number of genetic clusters (K) using the ΔK method; (**b**) STRUCTURE bar plots showing population genetic structure at K = 2. Each vertical bar represents one individual, and colors indicate membership coefficients in the two inferred genetic clusters.

Bayesian clustering analysis in STRUCTURE (K = 1–9), evaluated using the ΔK criterion of Evanno et al. [46], indicated K = 2 as the most likely number of genetic clusters (ΔK = 765.377), indicating that the strongest genetic partition in the dataset is represented by two major clusters (Figure 5). A much smaller secondary local peak was observed at K = 6 (ΔK = 19.099), suggesting the presence of additional fine-scale substructure within these two main groups (Figure 5a). At K = 2, individuals from Ketpentau, Sumbe, Kazachka, Butakovka and Pavlodar were assigned almost exclusively to cluster 1, forming a relatively homogeneous group. In contrast, most individuals from Almaty, Ile-Alatau, Koksu, and NorthKaz showed high membership coefficients for the second cluster. The Tekeli population was dominated by cluster 2 but also included a few individuals showing admixed ancestry, with partial membership in cluster 1. Overall, the STRUCTURE results indicate the presence of two main genetic clusters in the studied *L. dispar* populations, with only limited admixture in several localities (Figure 5b).

A much smaller secondary ΔK peak was observed at K = 6 (ΔK = 19.099), suggesting the possible presence of minor sub-structuring within the two main groups; however, because support for this partition was substantially lower than for K = 2, it was not interpreted further. Overall, the STRUCTURE results support the presence of two main genetic clusters among the studied *L. dispar* populations.

It should be noted that the interpretation of the observed genetic structure may to some extent depend on unequal sample sizes among geographic locations. In some populations, the number of individuals examined was relatively small, which could potentially reduce the stability of cluster assignments in the STRUCTURE analysis. In particular, small sample sizes may exhibit increased sensitivity to stochastic sampling effects and, consequently, partially biased estimates of the level of genetic admixture.

To quantify the observed patterns of genetic structure, a distance-based analysis of molecular variance (AMOVA) was performed using RAPD data. The results showed that 78.77% of the total genetic variation was distributed within populations, whereas 21.23% was attributed to differences among populations. The among-population component was statistically significant (*p* = 0.001), supporting the presence of genetic structuring consistent with the two broad nuclear groups observed in the clustering analyses, as shown in Table 3.

The overall level of genetic differentiation was further supported by the PhiPT value (0.212, *p* = 0.001), which indicates moderate to high differentiation among populations. Pairwise comparisons revealed heterogeneous levels of differentiation, with high PhiPT values observed between several population pairs (e.g., Koksu–Sumbe and Ketpentau–Koksu), while other comparisons showed low or non-significant differentiation, reflecting varying degrees of connectivity and gene flow across regions.

Overall, the RAPD-based analyses were broadly concordant: PCoA and STRUCTURE supported two partially overlapping nuclear genetic groups, while AMOVA confirmed significant among-population differentiation (ΦPT = 0.212, *p* = 0.001), indicating moderate but incomplete population structuring rather than complete separation of populations.

#### 3.3.2. Sequencing

Genetic diversity was also assessed using high-throughput sequencing of the COI amplicon. After filtering, the obtained reads matched *L. dispar* with approximately 99% sequence identity and mapping quality values exceeding 70%, covering most of the expected region, confirming the taxonomic identity of the analyzed specimens. Consensus calling produced 153 COI consensus sequences for downstream phylogenetic reconstruction.

**Figure 6:**
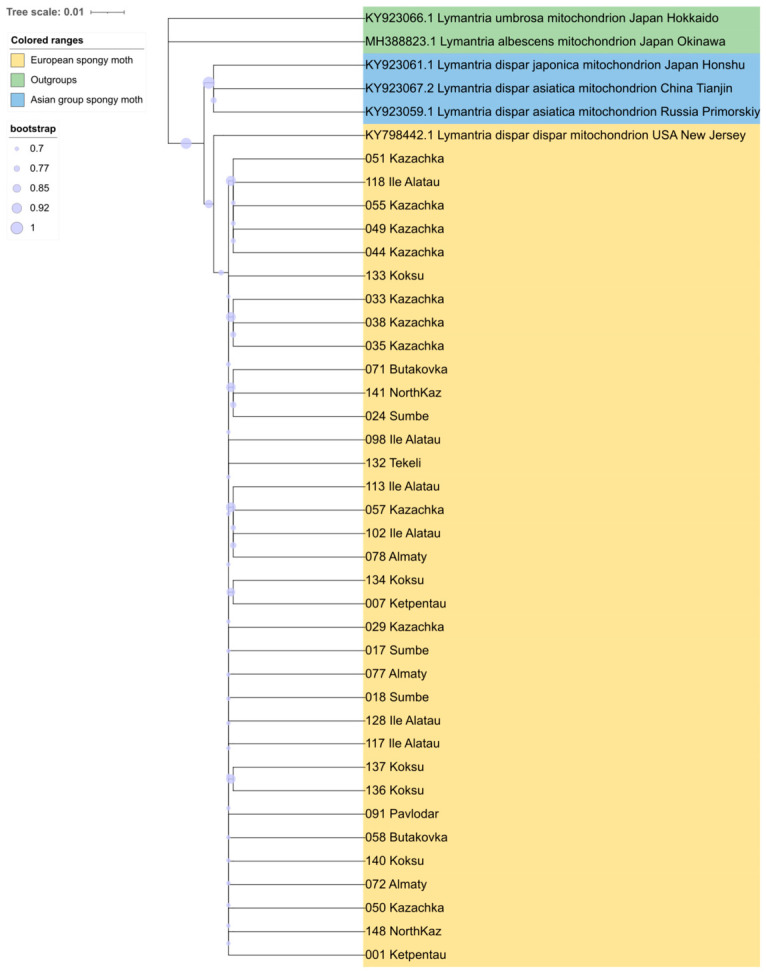
Phylogenetic relationships among *L. dispar* from Kazakhstan based on partial mitochondrial COI sequences.

Maximum-likelihood analysis of the COI dataset was performed using representative Kazakhstan sequences together with reference sequences of *Lymantria dispar dispar*, *L. d. asiatica*, *L. d. japonica*, and the outgroup taxa *Lymantria albescens* Hori & Umeno (Lepidoptera: Erebidae) and *Lymantria umbrosa* Butler (Lepidoptera: Erebidae). In the tree, the Kazakhstan specimens were placed within the *L. dispar* complex and formed a compact group closely associated with the sequence of *L. d. dispar*. The non-dispar taxa, *L. albescens* and *L. umbrosa*, were positioned outside the *L. dispar* group, supporting their use as outgroups (Figure 6).

The topology of the Kazakhstan sequences was characterized by very short internal branches, supported by moderate to high bootstrap values (0.74–0.99), indicating low mitochondrial divergence among the analyzed samples. No clear geographic clustering was observed among specimens from different localities. This pattern suggests that the COI marker does not resolve pronounced mitochondrial population structure within the Kazakhstan dataset.

COI phylogenetic analysis indicates that the Kazakhstan specimens share a relatively homogeneous mitochondrial background associated with *L. d. dispar*. However, the low COI variability and short internal branches suggest limited resolution of this marker for fine-scale population structure. Therefore, COI results should be interpreted together with nuclear markers, including FS1 and RAPD data.

## 4. Discussion

Understanding the genetic composition and population structure of *Lymantria dispar* populations is important for improving quarantine monitoring and management strategies, particularly in regions where different evolutionary lineages may come into contact. If substantial genetic differences exist among spongy moth populations in Kazakhstan, they may be associated with biologically important traits, including traits relevant to dispersal capacity, such as female flight capability, and may therefore require different diagnostic and management approaches. Previous studies in Central Asia have been geographically restricted or limited in scope, leaving a substantial gap in population-level genetic data [59,60,61]. By integrating mitochondrial COI markers, the nuclear FS1 locus, RAPD profiling, and COI sequencing, our study provides the first large-scale insight into the genetic structure of *L. dispar* populations in Kazakhstan.

Subspecies identification and detection of Asian introgression in this study were based on the widely used TaqMan assay developed by Stewart et al. [24], which combines mitochondrial COI markers with the nuclear FS1 locus. In our dataset, all specimens carried the European mitochondrial haplotype, whereas 92.889.5% of individuals exhibited Asian nuclear introgression at the FS1 locus. This finding may indicate that the classical concept of a strict east–west subspecies boundary, traditionally defined by the geographic separation of European and Asian forms of *Lymantria dispar*, is more complex than previously assumed and could reflect historical hybridization and gene flow [62]. Genome-wide analyses have demonstrated that *L. dispar* populations across Eurasia form a continuous genetic gradient rather than discrete subspecies units, reflecting extensive admixture and historical connectivity [22].

The lack of strict congruence between RAPD structure and geographic proximity suggests that factors other than isolation by distance may have contributed to the observed nuclear pattern. This was particularly evident in both southern and northern Kazakhstan, where geographically close populations were assigned to different RAPD groups. This pattern suggests that the observed nuclear structure is unlikely to result solely from isolation by distance. Instead, it may reflect a combination of historical introgression of Asian-associated nuclear alleles [22,23,24], local founder effects, heterogeneous gene flow, and human-mediated dispersal [63,64,65]. In *L. dispar*, egg masses may be transported over long distances on wood, vehicles, nursery stock, outdoor equipment, and other substrates, which can promote the movement of genotypes independently of geographic proximity. Such processes may be particularly relevant in Kazakhstan, where urban, peri-urban, forest, and transport-connected localities may differ in both natural population continuity and opportunities for anthropogenic movement. Therefore, the two RAPD-based clusters should not be interpreted as discrete subspecies or as a simple latitudinal division, but rather as evidence of moderate nuclear structuring within a complex contact zone. Because RAPD markers provide dominant multilocus profiles with limited genomic resolution, this interpretation should be considered preliminary and should be further tested using higher-resolution nuclear markers, such as microsatellites or genome-wide SNPs [39,66,67].

Our results further indicate that Kazakhstan represents a broad contact zone between European and Asian lineages. This interpretation is consistent with previous studies demonstrating that *L. dispar* populations across Eurasia exhibit extensive gene flow and admixture rather than discrete genetic boundaries. For example, mitochondrial analyses across Eurasia have shown that subspecies are not strictly monophyletic and may overlap due to historical and ongoing dispersal processes [3]. These findings support the idea that Central Asia functions as a transition zone with a complex genetic structure shaped by hybridization.

The combined analysis of RAPD and mitochondrial COI markers provides important insights into the genetic diversity of *L. dispar* populations in Kazakhstan. RAPD profiling revealed a relatively high level of nuclear genetic variability, as indicated by 107 polymorphic loci, partial clustering in PCoA, and the identification of two main genetic groups in STRUCTURE analysis. In contrast to the mitochondrial COI data, the RAPD-based ordination and clustering approaches revealed a clear distinction between two broad nuclear genetic groups, although this subdivision was not fully explained by the geographic position of the sampled populations. The AMOVA results demonstrated that a substantial and statistically significant proportion of genetic variation (21.23%, *p* = 0.001) is attributable to differences among populations. The corresponding ΦPT value (0.212, *p* = 0.001) further indicates moderate to high genetic differentiation, suggesting that population subdivision is more pronounced than implied by visual clustering alone. At the same time, the majority of genetic variation (78.77%) was found within populations, indicating considerable gene flow and admixture across regions. This combination of significant among-population differentiation and high within-population variability reflects a mosaic population structure, where partially differentiated populations coexist with genetically mixed groups. Such patterns are characteristic of systems with ongoing dispersal and secondary contact. Similar patterns have been reported in other studies using multilocus markers, where nuclear genetic variation is present but reflects gradual differentiation and connectivity rather than strong population isolation. For example, AFLP-based analyses demonstrated that although genetic differences among populations can be detected, they tend to follow a pattern of geographic continuity rather than forming discrete, isolated units [68]. Similarly, ISSR-based studies in Asian populations revealed high levels of polymorphism and moderate differentiation, with genetic structure largely reflecting geographic gradients rather than strict population boundaries [69]. More recent genome-wide analyses further support this interpretation, showing that *Lymantria dispar* populations across Eurasia form a continuous genetic cline characterized by admixture and gene flow rather than sharply separated subspecies or populations [22].

In contrast, mitochondrial COI data revealed extremely low genetic diversity, with all Kazakhstan samples forming a single, highly homogeneous clade characterized by minimal sequence divergence and extensive haplotype sharing across geographically distant regions. This pattern is consistent with findings from European populations, where mitochondrial COI analyses revealed low genetic diversity, limited population structure, and evidence of strong gene flow among populations [5]. Such results are often interpreted as evidence of recent population expansion or ongoing dispersal processes maintaining genetic homogeneity.

Comparable mitochondrial patterns have also been observed in Asian populations [29]. For example, a large-scale COI study demonstrated haplotype sharing across distant regions and genetic affinity between Asian and European populations, likely driven by dispersal and anthropogenic movement [29]. Importantly, some Chinese populations demonstrated genetic similarity to European lineages, further supporting the absence of strict boundaries between Eurasian groups and reinforcing the concept of a continuous genetic cline across Eurasia. Kang et al., using a combination of microsatellite and mitochondrial markers, revealed moderate nuclear differentiation while maintaining evidence of substantial genetic exchange among East Asian populations [14]. These findings further support the conclusion that mitochondrial markers alone may underestimate biologically meaningful genetic variation in *L. dispar*.

The contrast between relatively high nuclear variability and low mitochondrial diversity highlights pronounced mito-nuclear discordance in Kazakhstan populations. This pattern is consistent with scenarios of secondary contact and hybridization, where nuclear genomes diversify through recombination while mitochondrial lineages remain conserved. Similar discordance has been widely reported and is often associated with mitochondrial introgression and incomplete lineage sorting [70]. In particular, mito-nuclear discordance has been shown to arise following introgressive hybridization in zones of secondary contact [71]. Empirical studies demonstrate that mitochondrial data may indicate admixture, whereas nuclear markers retain clearer signals of population structure [72]. In insects, shared mitochondrial haplotypes among distinct lineages have been explained by repeated introgression events, whereas nuclear markers provide higher-resolution differentiation [73]. Such discordance is now recognized as a widespread phenomenon across animal taxa and reflects differences in inheritance patterns and evolutionary dynamics between mitochondrial and nuclear genomes [74].

One plausible explanation for the observed genetic patterns is the combined effect of natural dispersal and human-mediated movement. Anthropogenic transport has been shown to facilitate gene flow and mixing of previously isolated populations, contributing to the spread of genetic variants across large geographic distances [5]. The widespread occurrence of Asian nuclear alleles in the absence of Asian mitochondrial haplotypes observed in our study is consistent with the concept of introgressive hybridization previously discussed for Central Asian populations. Earlier studies demonstrated that populations from Kazakhstan and neighboring regions may contain mosaic genomes combining European mitochondrial lineages with Asian nuclear components [75]. The authors associated this pattern with secondary contact between previously diverged lineages followed by hybridization, which closely corresponds to the results obtained in our study. In addition, a global phylogeographic analysis of *L. dispar* confirmed the presence of a complex system of overlapping phylogeographic lineages and the absence of clear discrete boundaries between subspecies.

From the perspective of invasion biology, the detected patterns have important applied implications. High within-population variability combined with moderate among-population differentiation may increase the adaptive potential of populations and facilitate the successful colonization of new territories [23]. This is particularly important for quarantine management, since admixed and introgressed populations complicate the reconstruction of introduction pathways and the identification of invasion sources. A similar issue has been discussed in studies on biosurveillance of the Asian spongy moth, where mitochondrial markers were shown not always to reflect the complex structure of populations and may display discordance with nuclear data [59]. In particular, a recent study of Korean populations demonstrated that, despite low nucleotide diversity of COI, evidence of anthropogenically mediated haplotype dispersal could still be detected, likely associated with international trade and maritime transport [76].

Overall, our findings demonstrate that genetic diversity in Kazakhstan populations of *L. dispar* is better reflected by nuclear markers than by mitochondrial COI alone. The observed combination of low mitochondrial divergence, moderate nuclear structuring, and extensive introgression supports the interpretation of Kazakhstan as an active contact zone shaped by gene flow and hybridization. These results highlight the importance of multilocus approaches for accurate population characterization and underscore the need to incorporate nuclear markers into routine phytosanitary surveillance and risk-assessment programs.

## 5. Conclusions

This study offers the first comprehensive molecular–genetic analysis of spongy moth populations across Kazakhstan. It shows that this region functions as a broad contact zone within the larger *L. dispar dispar*–*L. dispar asiatica* gradient. Morphological analysis, subspecies-specific real-time PCR, RAPD profiling, and COI sequencing consistently indicate that all examined specimens are *L. dispar dispar* at the mitochondrial level. However, 92.889.5% of these individuals carry the Asian FS1 allele. This significant mito–nuclear discordance suggests extensive and long-term introgression of Asian nuclear alleles into populations that primarily have a European mitotype. It also questions the idea of a clear, sharp subspecies boundary in Central Asia.

RAPD-based multi-locus genotypes identified two main nuclear clusters aligned along a north–south axis, with significant mixing in several locations, especially in Almaty and Tekeli. Overall, there is weak to moderate differentiation, extensive sharing of polymorphisms, and no distinct mitochondrial lineages, which all indicate widespread gene flow driven both by natural dispersal and human activities.

From an applied standpoint, these findings highlight the need to include molecular diagnostics in routine phytosanitary surveillance of *L. dispar* in Kazakhstan. The widespread presence of Asian nuclear alleles in primarily European populations suggests a higher risk of traits linked to the Asian subspecies, such as increased dispersal ability and a wider host range. Ongoing monitoring that combines subspecies-specific real-time PCR assays with genome-wide nuclear markers and more detailed spatial sampling will be crucial to understanding the dynamics of introgression, improving risk assessments, and guiding evidence-based management strategies for this quarantine pest in Central Asia and beyond.

## Figures and Tables

**Table 1:** Location of collected samples.

Location	Coordinates	Date	Number of Samples
(A) Ketpentau, Kegen District, Almaty Region, Republic of Kazakhstan	43.296389704; 79.515002376	28 June 2023	16
(B) Sumbe, Kegen District, Almaty Region, Republic of Kazakhstan	43.289167482; 79.477224598	28 June 2023	11
(C) Kazachka Gorge, Bostandyk District, Almaty Region, Republic of Kazakhstan	43.125278643; 76.920557945	1 July 2023	29
(D) Butakovka Gorge, Medeu District, Almaty Region Republic of Kazakhstan	43.180834195; 77.085280168	24 June 2023	14
(E) Almaty City, Almaty region, Republic of Kazakhstan	43.222223085; 77.010835726	24 June 2023 12 July 2024	20
(F) Pavlodar City, Pavlodar Region, Republic of Kazakhstan	52.243056289; 76.964447213	28 June 2023 6 July 2024	6
(G) Ile-Alatau Mountains, Almaty Region, Republic of Kazakhstan	43.364167515; 77.680280170	19 June 2024	34
(H) Tekeli City, Jetisu Region, Republic of Kazakhstan	44.829167475; 78.824169103	28 June 2024	3
(I) Koksu Village, Koksu District, Jetisu Region, Republic of Kazakhstan	45.002778600; 77.947780227	6 August 2024	7
(J) Ayyrtau District, North Kazakhstan Region, Republic of Kazakhstan	52.648056405; 70.475836155	2 August 2024	13

**Table 2:** Predicted number of amplicons for each marker by in silico analysis.

Marker Title	Min Number	Max Number	1_1 * (Min–Max)	1_2 ** (Min–Max)	2_2 *** (Min–Max)
	Amplicons				
OPAN-03	1	8024	1–7	48–8024	471–1718
OPB-04	1	643	1–12	33–120	130–643
OPN-09	1	2029	1–16	71–684	198–2029
OPY-15	2	835	2–13	43–143	226–835
UBC378	5	1511	5–26	121–434	580–1511

* 1_1 exhibited a single nucleotide mismatch in both the forward and reverse primer binding regions; ** 1_2 showed one mismatch in one primer binding region (forward/reverse) and two mismatches in the opposite direction (reverse/forward); *** 2_2 contained two mismatches in both forward and reverse primer binding sites.

**Table 3:** Genetic diversity and population differentiation based on RAPD markers.

	Comparison	Statistic	Value	*p*-Value
AMOVA	Among populations	% variation	21.23	0.001
	Within populations	% variation	78.77	-
Overall differentiation	All populations	ΦPT	0.212	0.001
Pairwise differentiation	Koksu–Sumbe	ΦPT	0.328	<0.001
	Ketpentau–Koksu	ΦPT	0.289	<0.001
	NorthKaz–Sumbe	ΦPT	0.260	<0.001
	Almaty–Ketpentau	ΦPT	0.130	<0.01
	Almaty–NorthKaz	ΦPT	0.090	<0.05
	Almaty–Tekeli	ΦPT	-	ns

ns—non-significant.

## Data Availability

The sequencing data generated in this study are available in the OSF repository at https://doi.org/10.17605/OSF.IO/PAJ56. The assembled consensus sequences are available in GenBank under accession numbers PZ226911–PZ227064.

## Supplementary Materials

The following supporting information can be downloaded at: https://www.mdpi.com/article/10.3390/insects17060591/s1, Table S1: Experimental specimen information of *Lymantria dispar*; Table S2. RAPD marker electrophoresis images.

## Abbreviations

The following abbreviations are used in this manuscript:

RAPD	Randomly amplified polymorphic DNA
COI	Cytochrome oxidase
LNA	Locked nucleic acid
PCoA	Principal coordinates analysis
UPGMA	Unweighted pair group method with arithmetic mean
AMOVA	Analysis of molecular variance
MAFFT	Multiple alignment using fast Fourier transform
MEGA12	Molecular Evolutionary Genetics Analysis version 12

## References

[1] Boukouvala M.C., Kavallieratos N.G., Skourti A., Pons X., Alonso C.L., Eizaguirre M., Fernandez E.B., Solera E.D., Fita S., Bohinc T. (2022). *Lymantria dispar* (L.) (Lepidoptera: Erebidae): Current Status of Biology, Ecology, and Management in Europe with Notes from North America. Insects.

[2] Ji W., Dou F., Zhang C., Xiao Y., Yin W., Yu J., Kurenshchikov D.K., Zhu X., Shi J. (2023). Improvement in the Identification Technology for Asian Spongy Moth, *Lymantria dispar* Linnaeus, 1758 (Lepidoptera: Erebidae) Based on SS-COI. Insects.

[3] Zhao J., Wu Y., Kurenshchikov D.K., Ilyinykh A.V., Shi J. (2019). Underestimated Mitochondrial Diversity in Gypsy Moth *Lymantria dispar* from Asia. Agric. For. Entomol..

[4] Pogue M.G., Schaefer P.W. (2007). A Review of Selected Species of Lymantria Hübner (1819) (Lepidoptera: Noctuidae: Lymantriinae) from Subtropical and Temperate Regions of Asia, Including the Descriptions of Three New Species, Some Potentially Invasive to North America.

[5] Lacković N., Pernek M., Bertheau C., Franjević D., Stauffer C., Avtzis D.N. (2018). Limited Genetic Structure of Gypsy Moth Populations Reflecting a Recent History in Europe. Insects.

[6] Tobin P. (2013). Lymantria Dispar (Gypsy Moth). CABI Compend..

[7] Tobin P.C. (2015). Ecological Consequences of Pathogen and Insect Invasions. Curr. For. Rep..

[8] Liebhold A.M., Halverson J.A., Elmes G.A. (1992). Gypsy Moth Invasion in North America: A Quantitative Analysis. J. Biogeogr..

[9] Elkinton J.S., Liebhold A.M. (1990). Population Dynamics of Gypsy Moth in North America. Annu. Rev. Entomol..

[10] Areces-Berazain F. (2022). Lymantria Dispar Asiatica (Asian Gypsy Moth). CABI Compend..

[11] Ananko G.G., Kolosov A.V., Martemyanov V.V. (2022). Rock Microhabitats Provide Suitable Thermal Conditions for Overwintering Insects: A Case Study of the Spongy Moth (*Lymantria dispar* L.) Population in the Altai Mountains. Insects.

[12] Ananko G.G., Kolosov A.V. (2021). Asian Gypsy Moth (*Lymantria dispar* L.) Populations: Tolerance of Eggs to Extreme Winter Temperatures. J. Therm. Biol..

[13] Kurenshchikov D.K., Martemyanov V.V., Imranova E.L. (2020). Features of the Far Eastern Gypsy Moth (*Lymantria dispar* L.) Population Outbreak. Contemp. Probl. Ecol..

[14] Kang T.H., Han S.H., Lee H.S. (2017). Genetic Structure and Demographic History of *Lymantria dispar* (Linnaeus, 1758) (Lepidoptera: Erebidae) in Its Area of Origin and Adjacent Areas. Ecol. Evol..

[15] Liebhold A.M., Gottschalk K.W., Muzika R.-M., Montgomery M.E., Young R., O’Day K., Kelley B. (1995). Suitability of North American Tree Species to the Gypsy Moth: A Summary of Field and Laboratory Tests. Forest Service General Technical Report (Final).

[16] Simberloff D., Rejmanek M. (2011). Encyclopedia of Biological Invasions.

[17] Wallner W.E., McManus K.A. (1986). Proceedings, Lymantriidae: A Comparison of Features of New and Old World Tussock Moths.

[18] Trotter R.T., Limbu S., Hoover K., Nadel H., Keena M.A. (2020). Comparing Asian Gypsy Moth [*Lymantria dispar asiatica* (Lepidoptera: Erebidae) and *L. dispar japonica*] Trap Data From East Asian Ports with Lab Parameterized Phenology Models: New Tools and Questions. Ann. Entomol. Soc. Am..

[19] Wang Y., Harrison R.L., Shi J. (2021). Effects of Rearing Density on Developmental Traits of Two Different Biotypes of the Gypsy Moth, *Lymantria Dispar* L., from China and the USA. Insects.

[20] Wu Y., Du Q., Qin H., Shi J., Wu Z., Shao W. (2018). Rapid Identification of the Asian Gypsy Moth and Its Related Species Based on Mitochondrial DNA. Ecol. Evol..

[21] Zuo Y., Kurenshchikov D.K., Yu J., Zou Y., Wang Y., Wang Y., Shi J. (2019). Microsatellite and Morphological Analyses Reveal Unexpected Diversity in *Lymantria dispar* in China. Forests.

[22] Picq S., Wu Y., Martemyanov V.V., Pouliot E., Pfister S.E., Hamelin R., Cusson M. (2023). Range-Wide Population Genomics of the Spongy Moth, *Lymantria dispar* (Erebidae): Implications for Biosurveillance, Subspecies Classification and Phylogeography of a Destructive Moth. Evol. Appl..

[23] Wu Y., Molongoski J.J., Winograd D.F., Bogdanowicz S.M., Louyakis A.S., Lance D.R., Mastro V.C., Harrison R.G. (2015). Genetic Structure, Admixture and Invasion Success in a Holarctic Defoliator, the Gypsy Moth (*Lymantria dispar*, Lepidoptera: Erebidae). Mol. Ecol..

[24] Stewart D., Zahiri R., Djoumad A., Freschi L., Lamarche J., Holden D., Cervantes S., Ojeda D.I., Potvin A., Nisole A. (2016). A Multi-Species TaqMan PCR Assay for the Identification of Asian Gypsy Moths (*Lymantria* spp.) and Other Invasive Lymantriines of Biosecurity Concern to North America. PLoS ONE.

[25] Lymantria Dispar (LYMADI)[Categorization]| EPPO Global Database. https://gd.eppo.int/taxon/LYMADI/categorization.

[26] Savotikov I.F., Smetnik A.I., Orlinskii A.D. (1995). Situation of the Asian Form of Gypsy Moth (*Lymantria dispar*) in Russia and in the World. EPPO Bull..

[27] Ministry of Agriculture of the Republic of Kazakhstan On Approval of the List of Quarantine Objects and Alien Species in Relation to Which Plant Quarantine Measures and A list of Highly Dangerous Pests Are Established and Implemented. https://adilet.zan.kz/kaz/docs/V1500011739.

[28] EPPO Global Database Quarantine Lists of Kazakhstan (2017). https://gd.eppo.int/reporting/article-6263.

[29] Xu Y., Zhang S., Wang H., Wang M., Li G. (2019). Mitochondrial Gene Sequence (COI) Reveals the Genetic Structure and Demographic History of *Lymantria dispar* (Lepidoptera: Erebidae: Lymantriinae) in and around China. Insects.

[30] Marín D.V., Castillo D.K., López-Lavalle L.A.B., Chalarca J.R., Pérez C.R. (2021). An Optimized High-Quality DNA Isolation Protocol for *spodoptera frugiperda* J. E. Smith (Lepidoptera: Noctuidae). MethodsX.

[31] Ross K., Cooper N., Bidwell J.R., Elder J. (2002). Genetic Diversity and Metal Tolerance of Two Marine Species: A Comparison between Populations from Contaminated and Reference Sites. Mar. Pollut. Bull..

[32] Naik A., Prajapat P., Krishnamurthy R., Pathak J.M. (2017). Assessment of Genetic Diversity in Costus Pictus Accessions Based on RAPD and ISSR Markers. 3 Biotech..

[33] Akkir D., Budak Yıldıran F.A., Çakir Ş. (2009). Üç Yerli İpekböceği Irkının (Alaca, Bursa Beyazı ve Hatay Sarısı) RAPD-PCR ve SDS-PAGE Yöntemleri Ile Moleküler Analizi. Kafkas Univ. Vet. Fak. Derg..

[34] Baziar G., Jafari M., Noori M., Samarfard S. (2018). Evaluation of Genetic Diversity among Persian Fig Cultivars by Morphological Traits and RAPD Markers. HortScience.

[35] Wu L., Wu Y., Guo Q., Li S., Zhou K., Zhang J. (2011). Comparison of Genetic Diversity in Pogostemon Cablin from China Revealed by RAPD, Morphological and Chemical Analyses. J. Med. Plant Res..

[36] Konzen E., Peron R., Ito M., Brondani G., Tsai S. (2017). Molecular Identification of Bamboo Genera and Species Based on RAPD-RFLP Markers. Silva Fenn..

[37] Lencina K., Konzen E., Tsai S., Bisognin D. (2016). Genetic Analysis of Apuleia Leiocarpa as Revealed by Random Amplified Polymorphic DNA Markers: Prospects for Population Genetic Studies. Genet. Mol. Res..

[38] Tewari G., Singh I.J., Barat A. (2013). Population Structure Analysis of *Labeo gonius* from Three Reservoirs of Uttarakhand Using RAPD Marker. Curr. Sci..

[39] Zhao D., Wen L., Bi H., Zhu Z., Liu J., Zhang J., Shi Q., You H., Dong D., Liu Q. (2017). Genetic Diversity of Cucurbita Maxima Assessed Using Morphological Characteristics and Random-Amplified Polymorphic DNA Markers in China. Acta Agric. Scand. Sect. B—Soil Plant Sci..

[40] Xu Z., Bai J., Zhang Y., Li L., Min M., Cao J., Cao J., Xu Y., Li F., Ma L. (2023). Chromosome-Level Genome Assembly of the Asian Spongy Moths *Lymantria dispar asiatica*. Sci. Data.

[41] Rice P., Longden I., Bleasby A. (2000). EMBOSS: The European Molecular Biology Open Software Suite. Trends Genet..

[42] Hunter J. (2007). Matplotlib: A 2D Graphics Environment. Comput. Sci. Eng..

[43] McKinney W. Data Structures for Statistical Computing in Python. Proceedings of the 9th Python in Science Conference.

[44] Pritchard J.K., Stephens M., Donnelly P. (2000). Inference of Population Structure Using Multilocus Genotype Data. Genetics.

[45] Kopelman N.M., Mayzel J., Jakobsson M., Rosenberg N.A., Mayrose I. (2015). Clumpak: A Program for Identifying Clustering Modes and Packaging Population Structure Inferences across K. Mol. Ecol. Resour..

[46] Evanno G., Regnaut S., Goudet J. (2005). Detecting the Number of Clusters of Individuals Using the Software STRUCTURE: A Simulation Study. Mol. Ecol..

[47] Dixon P. (2003). VEGAN, a Package of R Functions for Community Ecology. J. Veg. Sci..

[48] Oksanen J., Simpson G.L., Blanchet F.G., Kindt R., Legendre P., Minchin P.R., O’Hara R.B., Solymos P., Stevens M.H.H., Szoecs E. vegan: Community Ecology Package, version 2.7-3. https://CRAN.R-project.org/package=vegan.

[49] Peakall R., Smouse P.E. (2006). Genalex 6: Genetic Analysis in Excel. Population Genetic Software for Teaching and Research. Mol. Ecol. Notes.

[50] R Core Team R: A Language and Environment for Statistical Computing. https://www.scirp.org/reference/referencespapers?referenceid=3456808.

[51] Excoffier L., Smouse P.E., Quattro J.M. (1992). Analysis of Molecular Variance Inferred from Metric Distances among DNA Haplotypes: Application to Human Mitochondrial DNA Restriction Data. Genetics.

[52] Folmer O., Black M., Hoeh W., Lutz R., Vrijenhoek R. (1994). DNA Primers for Amplification of Mitochondrial Cytochrome c Oxidase Subunit I from Diverse Metazoan Invertebrates. Mol. Mar. Biol. Biotechnol..

[53] De Coster W., D’Hert S., Schultz D.T., Cruts M., Van Broeckhoven C. (2018). NanoPack: Visualizing and Processing Long-Read Sequencing Data. Bioinformatics.

[54] Okonechnikov K., Golosova O., Fursov M., the UGENE Team (2012). Unipro UGENE: A Unified Bioinformatics Toolkit. Bioinformatics.

[55] Katoh K., Standley D.M. (2013). MAFFT Multiple Sequence Alignment Software Version 7: Improvements in Performance and Usability. Mol. Biol. Evol..

[56] Tamura K., Nei M. (1993). Estimation of the Number of Nucleotide Substitutions in the Control Region of Mitochondrial DNA in Humans and Chimpanzees. Mol. Biol. Evol..

[57] deWaard J.R., Mitchell A., Keena M.A., Gopurenko D., Boykin L.M., Armstrong K.F., Pogue M.G., Lima J., Floyd R., Hanner R.H. (2010). Towards a Global Barcode Library for *Lymantria* (Lepidoptera: Lymantriinae) Tussock Moths of Biosecurity Concern. PLoS ONE.

[58] Novák V.J.A., Hrozinka F., Starý B. (1976). Atlas of Insects Harmful to Forest Trees.

[59] Orozumbekov A.A., Liebhold A.M., Ponomarev V.I., Tobin P.C. (2009). Gypsy Moth (Lepidoptera: Lymantriidae) in Central Asia. Am. Entomol..

[60] Djoumad A., Nisole A., Zahiri R., Freschi L., Picq S., Gundersen-Rindal D.E., Sparks M.E., Dewar K., Stewart D., Maaroufi H. (2017). Comparative Analysis of Mitochondrial Genomes of Geographic Variants of the Gypsy Moth, *Lymantria dispar*, Reveals a Previously Undescribed Genotypic Entity. Sci. Rep..

[61] Wu Y., Bogdanowicz S.M., Andres J.A., Vieira K.A., Wang B., Cossé A., Pfister S.E. (2020). Tracking Invasions of a Destructive Defoliator, the Gypsy Moth (Erebidae: *Lymantria dispar*): Population Structure, Origin of Intercepted Specimens, and Asian Introgression into North America. Evol. Appl..

[62] Srivastava V., Keena M.A., Maennicke G.E., Hamelin R.C., Griess V.C. (2021). Potential Differences and Methods of Determining Gypsy Moth Female Flight Capabilities: Implications for the Establishment and Spread in Novel Habitats. Forests.

[63] Bigsby K.M., Tobin P.C., Sills E.O. (2011). Anthropogenic Drivers of Gypsy Moth Spread. Biol. Invasions.

[64] Global Review of Forest Pests and Diseases. https://www.fao.org/4/i0640e/i0640e00.htm.

[65] Spongy Moth|Animal and Plant Health Inspection Service. https://www.aphis.usda.gov/plant-pests-diseases/spongy-moth.

[66] Lynch M., Milligan B.G. (1994). Analysis of Population Genetic Structure with RAPD Markers. Mol. Ecol..

[67] Random Amplified Polymorphic DNA (RAPD). https://www.ncbi.nlm.nih.gov/probe/docs/techrapd/.

[68] Reineke A., Karlovsky P., Zebitz C.P.W. (1999). Amplified Fragment Length Polymorphism Analysis of Different Geographic Populations of the Gypsy Moth, *Lymantria dispar* (Lepidoptera: Lymantriidae). Bull. Entomol. Res..

[69] Chen F., Shi J., Luo Y., Sun S., Pu M. (2013). Genetic Characterization of the Gypsy Moth from China (Lepidoptera, Lymantriidae) Using Inter Simple Sequence Repeats Markers. PLoS ONE.

[70] Quattrini A.M., Snyder K.E., Purow-Ruderman R., Seiblitz I.G.L., Hoang J., Floerke N., Ramos N.I., Wirshing H.H., Rodriguez E., McFadden C.S. (2023). Mito-Nuclear Discordance within Anthozoa, with Notes on Unique Properties of Their Mitochondrial Genomes. Sci. Rep..

[71] Blasco-Aróstegui J., Simone Y., Paulo O.S., Prendini L. (2025). Mito-Nuclear Discordance Reveals Introgressive Hybridization Following Vicariance and Secondary Contact in Iberian Scorpions (Buthidae: *Buthus*). BMC Ecol. Evol..

[72] Dong X., Zhang H., Zhu X., Wang K., Xue H., Ye Z., Zheng C., Bu W. (2023). Mitochondrial Introgression and Mito-Nuclear Discordance Obscured the Closely Related Species Boundaries in *Cletus* Stål from China (Heteroptera: Coreidae). Mol. Phylogenetics Evol..

[73] Lužáková I., Kúdelová T., Krčmárik S., Kúdela M. (2026). Strong Mito-Nuclear Discordance and Sharing of Mitochondrial DNA among Species of the Simulium Variegatum Group (Diptera, Simuliidae) in Europe. Hydrobiologia.

[74] Toews D.P.L., Brelsford A. (2012). The Biogeography of Mitochondrial and Nuclear Discordance in Animals. Mol. Ecol..

[75] Zahiri R., Christian Schmidt B., Schintlmeister A., Yakovlev R.V., Rindoš M. (2019). Global Phylogeography Reveals the Origin and the Evolutionary History of the Gypsy Moth (Lepidoptera, Erebidae). Mol. Phylogenetics Evol..

[76] Bae J., Byun H.-M., Choi S., Jang G., Kang M., Kim E., Park J., Lee H.-S., Jung S. (2025). The Genetic Diversity of the Asian Spongy Moth, *Lymantria dispar asiatica* Vnukovskii (Lepidoptera: Erebidae), in Korea Based on Mitochondrial COI Analysis. Insects.

