# Supplementary material for: Genetic Variation of Spongy Moth (Lymantria dispar) in Kazakhstan
Source: Insects. 2026 Jun 4;17(6):591. doi: 10.3390/insects17060591 (PMC13301321; doi:10.3390/insects17060591)
Supplement: Supplementary file 1 [file insects-17-00591-s001.zip › Table S1.pdf]

Supplementary Materials

Genetic Variation of Spongy Moth (*Lymantria dispar*) in Kazakhstan

Alibek Makhambetov [1,2,†], Zarina Dairbekova [1,†], Bakyt Dulat [1,3], Abay Sagitov [1,3], Alexandr Pozharskiy [1], Yerlan Kydyrbayev [4], Allah Bakhsh [5] and Dilyara Gritsenko [1,2,3,*]

[1] Laboratory of Molecular Biology, Institute of Plant Biology and Biotechnology, Almaty 050040, Kazakhstan; alibekmahambetov@gmail.com (A.M.); dairbekovaz2001@gmail.com (Z.D.); bahytalt99@gmail.com (B.D.); a_sagitov@mail.ru (A.S.); aspozharsky@gmail.com (A.P.)
[2] Department of Molecular Biology and Genetics, Al-Farabi Kazakh National University, Almaty 050040, Kazakhstan
[3] Research Center AgriBioTech, Almaty 050040, Kazakhstan
[4] Institute of Biology, National Academy of Sciences of the Kyrgyz Republic, Bishkek 720071, Kyrgyzstan; yerlankydyrbay@gmail.com
[5] Centre of Excellence in Molecular Biology, University of the Punjab, Lahore 54000, Pakistan; abthebest@gmail.com
* Correspondence: d.gritsenko@ipbb.kz
† These authors contributed equally to this work.

Table S1. Experimental specimen information of *Lymantria dispar*.

Population	Sample	Location	Coordinates	Date	Life stage	Host
Ketpentau	1	Ketpentau Mountains, Kegen District, Almaty Region, Republic of Kazakhstan	43°17'47''N 79°30'54''E	28.06.2023	Larva	Barberry
	2	Ketpentau Mountains, Kegen District, Almaty Region, Republic of Kazakhstan	43°17'47''N 79°30'54''E	28.06.2023	Larva	Barberry
	3	Ketpentau Mountains, Kegen District, Almaty Region, Republic of Kazakhstan	43°17'47''N 79°30'54''E	28.06.2023	Larva	Barberry
	4	Ketpentau Mountains, Kegen District, Almaty Region, Republic of Kazakhstan	43°17'47''N 79°30'55''E	28.06.2023	Larva	Barberry
	5	Ketpentau Mountains, Kegen District, Almaty Region, Republic of Kazakhstan	43°17'47''N 79°30'55''E	28.06.2023	Larva	Barberry
	6	Ketpentau Mountains, Kegen District, Almaty Region, Republic of Kazakhstan	43°17'47''N 79°30'55''E	28.06.2023	Larva	Barberry
	7	Ketpentau Mountains, Kegen District, Almaty Region, Republic of Kazakhstan	43°17'47''N 79°30'55''E	28.06.2023	Larva	Barberry
	8	Ketpentau Mountains, Kegen District, Almaty Region, Republic of Kazakhstan	43°17'47''N 79°30'55''E	28.06.2023	Larva	Barberry
	9	Ketpentau Mountains, Kegen District, Almaty Region, Republic of Kazakhstan	43°17'47''N 79°30'56''E	28.06.2023	Larva	Apple

	10	Ketpentau Mountains, Kegen District, Almaty Region, Republic of Kazakhstan	43°17'47"N 79°30'56"E	28.06.2023	Larva	Apple
	11	Ketpentau Mountains, Kegen District, Almaty Region, Republic of Kazakhstan	43°17'47"N 79°30'56"E	28.06.2023	Larva	Apple
	12	Ketpentau Mountains, Kegen District, Almaty Region, Republic of Kazakhstan	43°17'48"N 79°30'56"E	28.06.2023	Larva	Apple
	13	Ketpentau Mountains, Kegen District, Almaty Region, Republic of Kazakhstan	43°17'48"N 79°30'55"E	28.06.2023	Larva	Apple
	14	Ketpentau Mountains, Kegen District, Almaty Region, Republic of Kazakhstan	43°17'49"N 79°30'54"E	28.06.2023	Larva	Apple
	15	Ketpentau Mountains, Kegen District, Almaty Region, Republic of Kazakhstan	43°10'58"N 77°04'57"E	28.06.2023	Larva	Apple
	16	Ketpentau Mountains, Kegen District, Almaty Region, Republic of Kazakhstan	43°10'58"N 77°04'57"E	28.06.2023	Larva	Apple
Sumbe	17	Sumbe, Kegen District, Almaty Region, Republic of Kazakhstan	43°17'21"N 79°28'38"E	28.06.2023	Larva	Apple
	18	Sumbe, Kegen District, Almaty Region, Republic of Kazakhstan	43°17'21"N 79°28'38"E	28.06.2023	Larva	Apple
	19	Sumbe, Kegen District, Almaty Region, Republic of Kazakhstan	43°17'22"N 79°28'50"E	28.06.2023	Larva	Barberry
	20	Sumbe, Kegen District, Almaty Region, Republic of Kazakhstan	43°17'22"N 79°28'51"E	28.06.2023	Larva	Apple
	21	Sumbe, Kegen District, Almaty Region, Republic of Kazakhstan	43°17'23"N 79°28'45"E	28.06.2023	Larva	Apple
	22	Sumbe, Kegen District, Almaty Region, Republic of Kazakhstan	43°17'21"N 79°28'39"E	28.06.2023	Larva	Apple
	23	Sumbe, Kegen District, Almaty Region, Republic of Kazakhstan	43°17'22"N 79°28'49"E	28.06.2023	Larva	Apple
	24	Sumbe, Kegen District, Almaty Region, Republic of Kazakhstan	43°17'22"N 79°28'48"E	28.06.2023	Larva	Barberry
	25	Sumbe, Kegen District, Almaty Region, Republic of Kazakhstan	43°17'22"N 79°28'47"E	28.06.2023	Larva	Barberry
	26	Sumbe, Kegen District, Almaty Region, Republic of Kazakhstan	43°17'22"N 79°28'46"E	28.06.2023	Larva	Apple
	27	Sumbe, Kegen District, Almaty Region, Republic of Kazakhstan	43°17'22"N 79°28'45"E	28.06.2023	Larva	Apple
Kazachka	28	Kazachka Gorge, Bostandyk District, Almaty City, Republic of Kazakhstan	43°07'31"N 76°55'14"E	01.07.2023	Larva	Hawthorn
	29	Kazachka Gorge, Bostandyk District, Almaty City, Republic of Kazakhstan	43°07'30"N 76°55'14"E	01.07.2023	Larva	Barberry
	30	Kazachka Gorge, Bostandyk District, Almaty City, Republic of Kazakhstan	43°07'30"N 76°55'14"E	01.07.2023	Larva	Barberry
	31	Kazachka Gorge, Bostandyk District, Almaty City, Republic of Kazakhstan	43°07'30"N 76°55'14"E	01.07.2023	Larva	Apple

	32	Kazachka Gorge, Bostandyk District, Almaty City, Republic of Kazakhstan	43°07'30"N 76°55'15"E	01.07.2023	Larva	Hawthorn
	33	Kazachka Gorge, Bostandyk District, Almaty City, Republic of Kazakhstan	43°07'30"N 76°55'15"E	01.07.2023	Larva	Barberry
	34	Kazachka Gorge, Bostandyk District, Almaty City, Republic of Kazakhstan	43°07'30"N 76°55'15"E	01.07.2023	Larva	Hawthorn
	35	Kazachka Gorge, Bostandyk District, Almaty City, Republic of Kazakhstan	43°07'30"N 76°55'15"E	01.07.2023	Larva	Barberry
	36	Kazachka Gorge, Bostandyk District, Almaty City, Republic of Kazakhstan	43°07'30"N 76°55'16"E	01.07.2023	Larva	Hawthorn
	37	Kazachka Gorge, Bostandyk District, Almaty City, Republic of Kazakhstan	43°07'30"N 76°55'17"E	01.07.2023	Larva	Hawthorn
	38	Kazachka Gorge, Bostandyk District, Almaty City, Republic of Kazakhstan	43°07'30"N 76°55'17"E	01.07.2023	Larva	Barberry
	39	Kazachka Gorge, Bostandyk District, Almaty City, Republic of Kazakhstan	43°07'30"N 76°55'18"E	01.07.2023	Larva	Hawthorn
	40	Kazachka Gorge, Bostandyk District, Almaty City, Republic of Kazakhstan	43°07'30"N 76°55'18"E	01.07.2023	Larva	Barberry
	41	Kazachka Gorge, Bostandyk District, Almaty City, Republic of Kazakhstan	43°07'31"N 76°55'19"E	01.07.2023	Larva	Hawthorn
	42	Kazachka Gorge, Bostandyk District, Almaty City, Republic of Kazakhstan	43°07'31"N 76°55'19"E	01.07.2023	Larva	Hawthorn
	43	Kazachka Gorge, Bostandyk District, Almaty City, Republic of Kazakhstan	43°07'31"N 76°55'20"E	01.07.2023	Larva	Apple
	44	Kazachka Gorge, Bostandyk District, Almaty City, Republic of Kazakhstan	43°07'31"N 76°55'20"E	01.07.2023	Larva	Hawthorn
	45	Kazachka Gorge, Bostandyk District, Almaty City, Republic of Kazakhstan	43°07'31"N 76°55'21"E	01.07.2023	Larva	Hawthorn
	46	Kazachka Gorge, Bostandyk District, Almaty City, Republic of Kazakhstan	43°07'30"N 76°55'12"E	01.07.2023	Larva	Barberry
	47	Kazachka Gorge, Bostandyk District, Almaty City, Republic of Kazakhstan	43°07'30"N 76°55'12"E	01.07.2023	Larva	Apple
	48	Kazachka Gorge, Bostandyk District, Almaty City, Republic of Kazakhstan	43°07'29"N 76°55'11"E	01.07.2023	Larva	Barberry
	49	Kazachka Gorge, Bostandyk District, Almaty City, Republic of Kazakhstan	43°07'29"N 76°55'11"E	01.07.2023	Larva	Barberry

	50	Kazachka Gorge, Bostandyk District, Almaty City, Republic of Kazakhstan	43°07'29"N 76°55'11"E	01.07.2023	Larva	Hawthorn
	51	Kazachka Gorge, Bostandyk District, Almaty City, Republic of Kazakhstan	43°07'29"N 76°55'10"E	01.07.2023	Larva	Barberry
	52	Kazachka Gorge, Bostandyk District, Almaty City, Republic of Kazakhstan	43°07'29"N 76°55'10"E	01.07.2023	Larva	Apple
	53	Kazachka Gorge, Bostandyk District, Almaty City, Republic of Kazakhstan	43°07'28"N 76°55'11"E	01.07.2023	Larva	Rosehip
	54	Kazachka Gorge, Bostandyk District, Almaty City, Republic of Kazakhstan	43°07'28"N 76°55'11"E	01.07.2023	Larva	Apple
	55	Kazachka Gorge, Bostandyk District, Almaty City, Republic of Kazakhstan	43°07'28"N 76°55'10"E	01.07.2023	Larva	Apple
	56	Kazachka Gorge, Bostandyk District, Almaty City, Republic of Kazakhstan	43°07'28"N 76°55'10"E	01.07.2023	Larva	Barberry
Butakovka	57	Butakovka Gorge, Medeu District, Almaty City, Republic of Kazakhstan	43°10'51"N 77°05'07"E	24.06.2023	Larva	Hawthorn
	58	Butakovka Gorge, Medeu District, Almaty City, Republic of Kazakhstan	43°10'51"N 77°05'07"E	24.06.2023	Larva	Hawthorn
	59	Butakovka Gorge, Medeu District, Almaty City, Republic of Kazakhstan	43°10'56"N 77°05'06"E	24.06.2023	Larva	Hawthorn
	60	Butakovka Gorge, Medeu District, Almaty City, Republic of Kazakhstan	43°10'56"N 77°05'06"E	24.06.2023	Larva	Hawthorn
	61	Butakovka Gorge, Medeu District, Almaty City, Republic of Kazakhstan	43°10'57"N 77°05'05"E	24.06.2023	Larva	Hawthorn
	62	Butakovka Gorge, Medeu District, Almaty City, Republic of Kazakhstan	43°10'57"N 77°05'02"E	24.06.2023	Larva	Hawthorn
	63	Butakovka Gorge, Medeu District, Almaty City, Republic of Kazakhstan	43°10'57"N 77°05'04"E	24.06.2023	Larva	Hawthorn
	64	Butakovka Gorge, Medeu District, Almaty City, Republic of Kazakhstan	43°10'57"N 77°05'03"E	24.06.2023	Larva	Apple
	65	Butakovka Gorge, Medeu District, Almaty City, Republic of Kazakhstan	43°10'57"N 77°05'03"E	24.06.2023	Larva	Apple
	66	Butakovka Gorge, Medeu District, Almaty City, Republic of Kazakhstan	43°10'57"N 77°05'02"E	24.06.2023	Larva	Apple
	67	Butakovka Gorge, Medeu District, Almaty City, Republic of Kazakhstan	43°10'57"N 77°05'02"E	24.06.2023	Larva	Apple

	68	Butakovka Gorge, Medeu District, Almaty City, Republic of Kazakhstan	43°10'56"N 77°05'01"E	24.06.2023	Larva	Apple
	69	Butakovka Gorge, Medeu District, Almaty City, Republic of Kazakhstan	43°10'56"N 77°05'00"E	24.06.2023	Larva	Hawthorn
	70	Butakovka Gorge, Medeu District, Almaty City, Republic of Kazakhstan	43°10'56"N 77°04'56"E	24.06.2023	Larva	Hawthorn
Almaty	71	Almaty City, Republic of Kazakhstan	43°13'20"N 77°00'39"E	24.06.2023	Larva	Apple
	72	Almaty City, Republic of Kazakhstan	43°13'20"N 77°00'39"E	24.06.2023	Larva	Apple
	73	Almaty City, Republic of Kazakhstan	43°13'19"N 77°00'40"E	24.06.2023	Larva	Apple
	74	Almaty City, Republic of Kazakhstan	43°13'19"N 77°00'40"E	24.06.2023	Larva	Apple
	75	Almaty City, Republic of Kazakhstan	43°13'19"N 77°00'40"E	24.06.2023	Larva	Apple
	76	Almaty City, Republic of Kazakhstan	43°13'19"N 77°00'38"E	24.06.2023	Larva	Apple
	77	Almaty City, Republic of Kazakhstan	43°13'20"N 77°00'41"E	24.06.2023	Larva	Apple
	78	Almaty City, Republic of Kazakhstan	43°12'09"N 76°59'38"E	12.07.2024	Imago female*	Apple
	79	Almaty City, Republic of Kazakhstan	43°12'09"N 76°59'38"E	12.07.2024	Imago female*	Apple
	80	Almaty City, Republic of Kazakhstan	43°12'09"N 76°59'38"E	12.07.2024	Imago female*	Apple
	81	Almaty City, Republic of Kazakhstan	43°12'04"N 76°59'23"E	12.07.2024	Imago female*	Apple
	82	Almaty City, Republic of Kazakhstan	43°12'04"N 76°59'23"E	12.07.2024	Imago male*	Hawthorn
	83	Almaty City, Republic of Kazakhstan	43°12'04"N 76°59'38"E	12.07.2024	Imago female*	Hawthorn
	84	Almaty City, Republic of Kazakhstan	43°12'04"N 76°59'23"E	12.07.2024	Imago male*	Hawthorn
	85	Almaty City, Republic of Kazakhstan	43°12'09"N 76°59'32"E	12.07.2024	Imago male*	Apple
	86	Almaty City, Republic of Kazakhstan	43°12'09"N 76°59'32"E	12.07.2024	Imago male*	Apple
	87	Almaty City, Republic of Kazakhstan	43°12'08"N 76°59'35"E	12.07.2024	Imago female*	Apple
	88	Almaty City, Republic of Kazakhstan	43°12'03"N 76°59'39"E	12.07.2024	Imago male*	Hawthorn
	89	Almaty City, Republic of Kazakhstan	43°12'03"N 76°59'39"E	12.07.2024	Imago male*	Apple
	90	Almaty City, Republic of Kazakhstan	43°12'03"N 76°59'39"E	12.07.2024	Imago male*	Apple
Pavlodar	91	Pavlodar City, Pavlodar Region, Republic of Kazakhstan	52°14'35"N 76°57'52"E	28.06.2023	Larva	Oak
	92	Terenkol Village, Terenkol District, Pavlodar Region, Republic of Kazakhstan	53°03'59"N 76°04'20"E	06.07.2024	Larva	Oak

	93	Aktogay Village, Aktogay District, Pavlodar Region, Republic of Kazakhstan	53°01'04"N 75°58'43"E	06.07.2024	Larva	Oak
	94	Yertis Village, Yertis District, Pavlodar Region, Republic of Kazakhstan	53°19'48"N 75°27'33"E	28.06.2023	Larva	Oak
	95	Sharbakty Village, Sharbakty District, Pavlodar Region, Republic of Kazakhstan	52°29'30"N 78°09'21"E	28.06.2023	Larva	Oak
	96	Zhelezinka Village, Zhelezin District, Pavlodar Region, Republic of Kazakhstan	53°32'23"N 75°17'05"E	28.06.2023	Larva	Oak
Ile-Alatau	97	Ile-Alatau Mountains, Almaty Region, Republic of Kazakhstan	43°21'51"N 77°40'49"E	19.06.2024	Larva	Apple
	98	Ile-Alatau Mountains, Almaty Region, Republic of Kazakhstan	43°21'51"N 77°40'49"E	19.06.2024	Larva	Apple
	99	Ile-Alatau Mountains, Almaty Region, Republic of Kazakhstan	43°21'50"N 77°40'48"E	19.06.2024	Larva	Apple
	100	Ile-Alatau Mountains, Almaty Region, Republic of Kazakhstan	43°21'50"N 77°40'47"E	19.06.2024	Larva	Apple
	101	Ile-Alatau Mountains, Almaty Region, Republic of Kazakhstan	43°21'50"N 77°40'47"E	19.06.2024	Larva	Apple
	102	Ile-Alatau Mountains, Almaty Region, Republic of Kazakhstan	43°21'50"N 77°40'46"E	19.06.2024	Larva	Apple
	103	Ile-Alatau Mountains, Almaty Region, Republic of Kazakhstan	43°21'50"N 77°40'46"E	19.06.2024	Larva	Apple
	104	Ile-Alatau Mountains, Almaty Region, Republic of Kazakhstan	43°21'50"N 77°40'46"E	19.06.2024	Larva	Apple
	105	Ile-Alatau Mountains, Almaty Region, Republic of Kazakhstan	43°21'49"N 77°40'46"E	19.06.2024	Larva	Apple
	106	Ile-Alatau Mountains, Almaty Region, Republic of Kazakhstan	43°21'50"N 77°40'45"E	19.06.2024	Larva	Apple
	107	Ile-Alatau Mountains, Almaty Region, Republic of Kazakhstan	43°21'50"N 77°40'45"E	19.06.2024	Larva	Apple
	108	Ile-Alatau Mountains, Almaty Region, Republic of Kazakhstan	43°21'50"N 77°40'47"E	19.06.2024	Larva	Apple
	109	Ile-Alatau Mountains, Almaty Region, Republic of Kazakhstan	43°21'50"N 77°40'47"E	19.06.2024	Larva	Apple
	110	Ile-Alatau Mountains, Almaty Region, Republic of Kazakhstan	43°21'51"N 77°40'44"E	19.06.2024	Larva	Apple
	111	Ile-Alatau Mountains, Almaty Region, Republic of Kazakhstan	43°21'51"N 77°40'44"E	19.06.2024	Larva	Apple
	112	Ile-Alatau Mountains, Almaty Region, Republic of Kazakhstan	43°21'51"N 77°40'45"E	19.06.2024	Larva	Apple
	113	Ile-Alatau Mountains, Almaty Region, Republic of Kazakhstan	43°21'51"N 77°40'45"E	19.06.2024	Larva	Apple
	114	Ile-Alatau Mountains, Almaty Region, Republic of Kazakhstan	43°21'52"N 77°40'43"E	19.06.2024	Larva	Apple
	115	Ile-Alatau Mountains, Almaty Region, Republic of Kazakhstan	43°21'52"N 77°40'43"E	19.06.2024	Larva	Apple
	116	Ile-Alatau Mountains, Almaty Region, Republic of Kazakhstan	43°21'52"N 77°40'43"E	19.06.2024	Larva	Apple
	117	Ile-Alatau Mountains, Almaty Region, Republic of Kazakhstan	43°21'53"N 77°40'43"E	19.06.2024	Larva	Apple
	118	Ile-Alatau Mountains, Almaty Region, Republic of Kazakhstan	43°21'53"N 77°40'43"E	19.06.2024	Larva	Apple

	119	Ile-Alatau Mountains, Almaty Region, Republic of Kazakhstan	43°21'52"N 77°40'49"E	19.06.2024	Larva	Apple
	120	Ile-Alatau Mountains, Almaty Region, Republic of Kazakhstan	43°21'51"N 77°40'49"E	19.06.2024	Larva	Apple
	121	Ile-Alatau Mountains, Almaty Region, Republic of Kazakhstan	43°21'51"N 77°40'49"E	19.06.2024	Larva	Apple
	122	Ile-Alatau Mountains, Almaty Region, Republic of Kazakhstan	43°21'51"N 77°40'49"E	19.06.2024	Larva	Apple
	123	Ile-Alatau Mountains, Almaty Region, Republic of Kazakhstan	43°21'51"N 77°40'49"E	19.06.2024	Larva	Apple
	124	Ile-Alatau Mountains, Almaty Region, Republic of Kazakhstan	43°21'49"N 77°40'47"E	19.06.2024	Larva	Apple
	125	Ile-Alatau Mountains, Almaty Region, Republic of Kazakhstan	43°21'49"N 77°40'47"E	19.06.2024	Larva	Apple
	126	Ile-Alatau Mountains, Almaty Region, Republic of Kazakhstan	43°21'49"N 77°40'47"E	19.06.2024	Larva	Apple
	127	Ile-Alatau Mountains, Almaty Region, Republic of Kazakhstan	43°21'51"N 77°40'44"E	19.06.2024	Larva	Apple
	128	Ile-Alatau Mountains, Almaty Region, Republic of Kazakhstan	43°21'51"N 77°40'44"E	19.06.2024	Larva	Apple
	129	Ile-Alatau Mountains, Almaty Region, Republic of Kazakhstan	43°21'51"N 77°40'44"E	19.06.2024	Larva	Apple
	130	Ile-Alatau Mountains, Almaty Region, Republic of Kazakhstan	43°21'51"N 77°40'44"E	19.06.2024	Imago female*	Apple
Tekeli	131	Tekeli City, Jetisu Region, Republic of Kazakhstan	44°49'45"N 78°49'27"E	28.06.2024	Larva	Apple
	132	Tekeli City, Jetisu Region, Republic of Kazakhstan	44°49'45"N 78°49'27"E	28.06.2024	Larva	Apple
	133	Tekeli City, Jetisu Region, Republic of Kazakhstan	44°49'45"N 78°49'27"E	28.06.2024	Imago female*	Apple
Koksu	134	Koksu Village, Koksu District, Jetisu Region, Republic of Kazakhstan	45°00'10"N 77°56'52"E	06.08.2024	Imago (male)	Oak
	135	Koksu Village, Koksu District, Jetisu Region, Republic of Kazakhstan	45°00'10"N 77°56'52"E	06.08.2024	Imago (male)	Oak
	136	Koksu Village, Koksu District, Jetisu Region, Republic of Kazakhstan	45°00'10"N 77°56'52"E	06.08.2024	Imago (male)	Oak
	137	Koksu Village, Koksu District, Jetisu Region, Republic of Kazakhstan	45°00'09"N 77°56'46"E	06.08.2024	Imago (male)	Oak
	138	Koksu Village, Koksu District, Jetisu Region, Republic of Kazakhstan	45°00'09"N 77°56'46"E	06.08.2024	Imago (male)	Oak
	139	Koksu Village, Koksu District, Jetisu Region, Republic of Kazakhstan	45°00'09"N 77°56'46"E	06.08.2024	Imago (male)	Oak
	140	Koksu Village, Koksu District, Jetisu Region, Republic of Kazakhstan	45°00'09"N 77°56'46"E	06.08.2024	Imago (male)	Oak
North Kazakhstan	141	Bulandy District, Akmola Region, Republic of Kazakhstan	52°38'53"N 70°28'33"E	02.08.2024	Imago (male)	Oak
	142	Zerendi District, Akmola Region, Republic of Kazakhstan	52°53'37"N 69°08'14"E	02.08.2024	Imago (male)	Oak

	143	Zerendi District, Akmola Region, Republic of Kazakhstan	52°53'37"N 69°08'15"E	02.08.2024	Imago (male)	Oak
	144	Sandyktau District, Akmola Region, Republic of Kazakhstan	52°36'21"N 68°52'03"E	02.08.2024	Imago (male)	Oak
	145	Sandyktau District, Akmola Region, Republic of Kazakhstan	52°36'21"N 68°52'01"E	02.08.2024	Imago (male)	Oak
	146	Sandyktau District, Akmola Region, Republic of Kazakhstan	52°36'21"N 68°52'00"E	02.08.2024	Imago (male)	Oak
	147	Akkol District, Akmola Region, Republic of Kazakhstan	51°59'51"N 70°54'56"E	25.07.2024	Imago (male)	Apple
	148	Akkol District, Akmola Region, Republic of Kazakhstan	51°59'51"N 70°54'56"E	25.07.2024	Imago (male)	Apple
	149	Astana City, Republic of Kazakhstan	51°09'34"N 71°21'47"E	02.08.2024	Imago (male)	Oak
	150	Astana City, Republic of Kazakhstan	51°09'34"N 71°21'47"E	02.08.2024	Imago (male)	Oak
	151	Astana City, Republic of Kazakhstan	51°09'34"N 71°21'47"E	02.08.2024	Imago (male)	Oak
	152	Astana City, Republic of Kazakhstan	51°09'34"N 71°21'48"E	02.08.2024	Imago (male)	Oak
	153	Ayyrtau District, North Kazakhstan Region, Republic of Kazakhstan	53°16'39"N 68°04'16"E	02.08.2024	Imago (male)	Oak

* Specimens marked with an asterisk were collected manually at the larval stage and maintained under controlled laboratory conditions at 25 °C and 64% relative humidity. After pupation, adults emerged under these conditions and were used for subsequent examination.
