# Supplementary material for: Genetic Variation of Spongy Moth (Lymantria dispar) in Kazakhstan
Source: Insects. 2026 Jun 4;17(6):591. doi: 10.3390/insects17060591 (PMC13301321; doi:10.3390/insects17060591)
Supplement: Supplementary file 1 [file insects-17-00591-s001.zip › Table S2.pdf]

Supplementary Materials

Genetic Variation of Spongy Moth (*Lymantria dispar*) in Kazakhstan

Alibek Makhambetov [1,2,†], Zarina Dairbekova [1,†], Bakyt Dulat [1,3], Abay Sagitov [1,3], Alexandr Pozharskiy [1], Yerlan Kydyrbayev [4], Allah Bakhsh [5] and Dilyara Gritsenko [1,2,3,*]

[1] Laboratory of Molecular Biology, Institute of Plant Biology and Biotechnology, Almaty 050040, Kazakhstan; alibekmahambetov@gmail.com (A.M.); dairbekovaz2001@gmail.com (Z.D.); bahytalt99@gmail.com (B.D.); a_sagitov@mail.ru (A.S.); aspozharsky@gmail.com (A.P.)
[2] Department of Molecular Biology and Genetics, Al-Farabi Kazakh National University, Almaty 050040, Kazakhstan
[3] Research Center AgriBioTech, Almaty 050040, Kazakhstan
[4] Institute of Biology, National Academy of Sciences of the Kyrgyz Republic, Bishkek 720071, Kyrgyzstan; yerlankydyrbay@gmail.com
[5] Centre of Excellence in Molecular Biology, University of the Punjab, Lahore 54000, Pakistan; abthebest@gmail.com
* Correspondence: d.gritsenko@ipbb.kz
† These authors contributed equally to this work.

Table S2. *RAPD marker electrophoresis images.*

Primer Names	Images
OPB-04	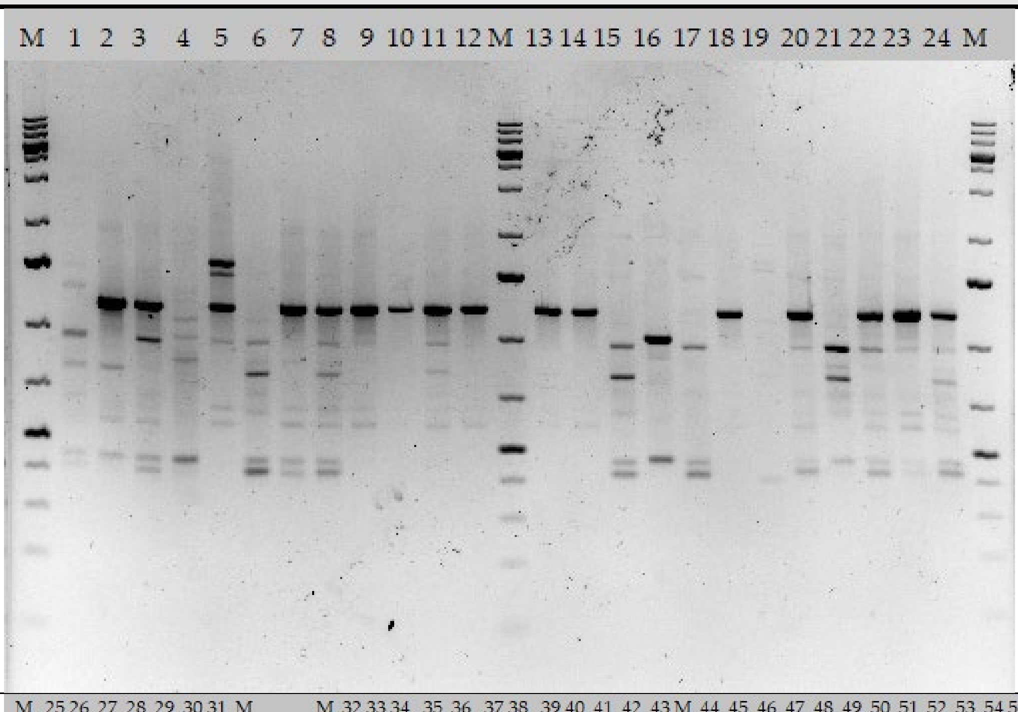 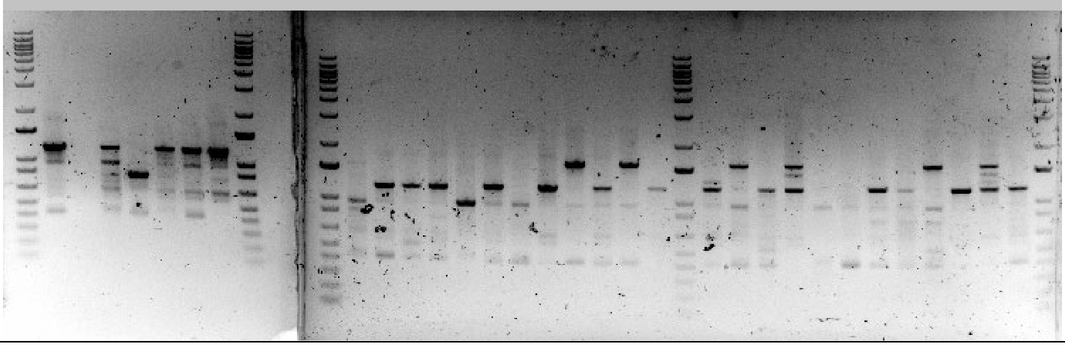

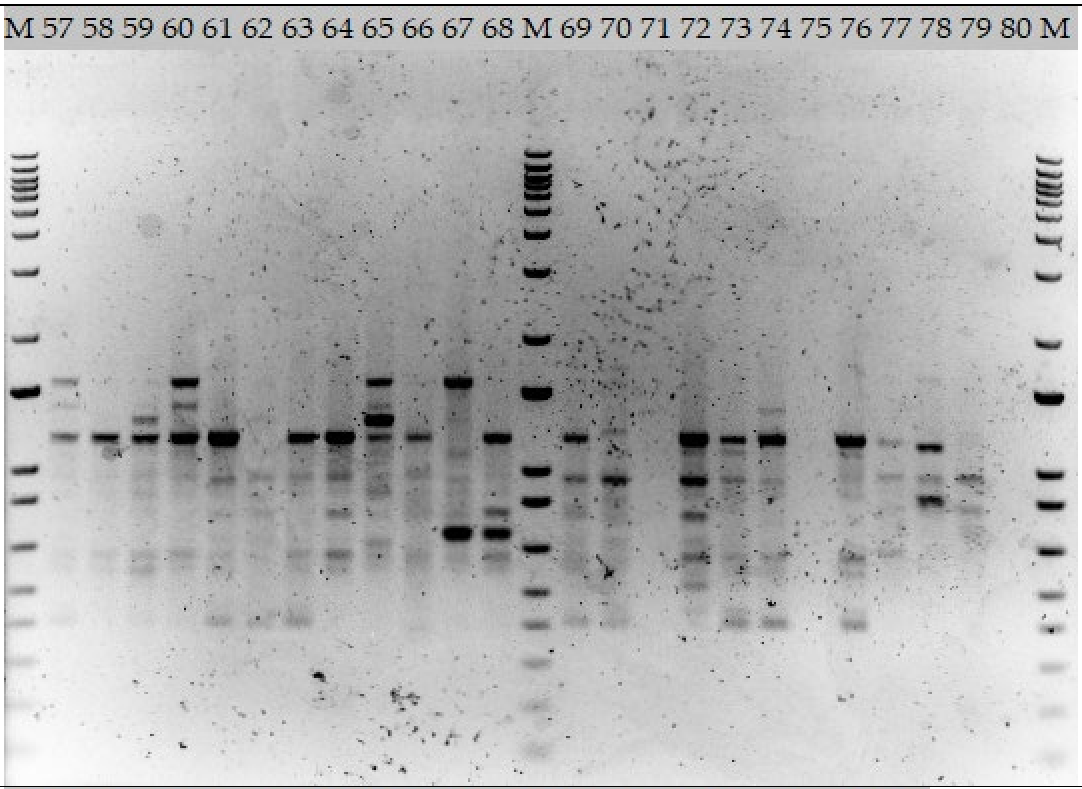
M 57 58 59 60 61 62 63 64 65 66 67 68 M 69 70 71 72 73 74 75 76 77 78 79 80 M

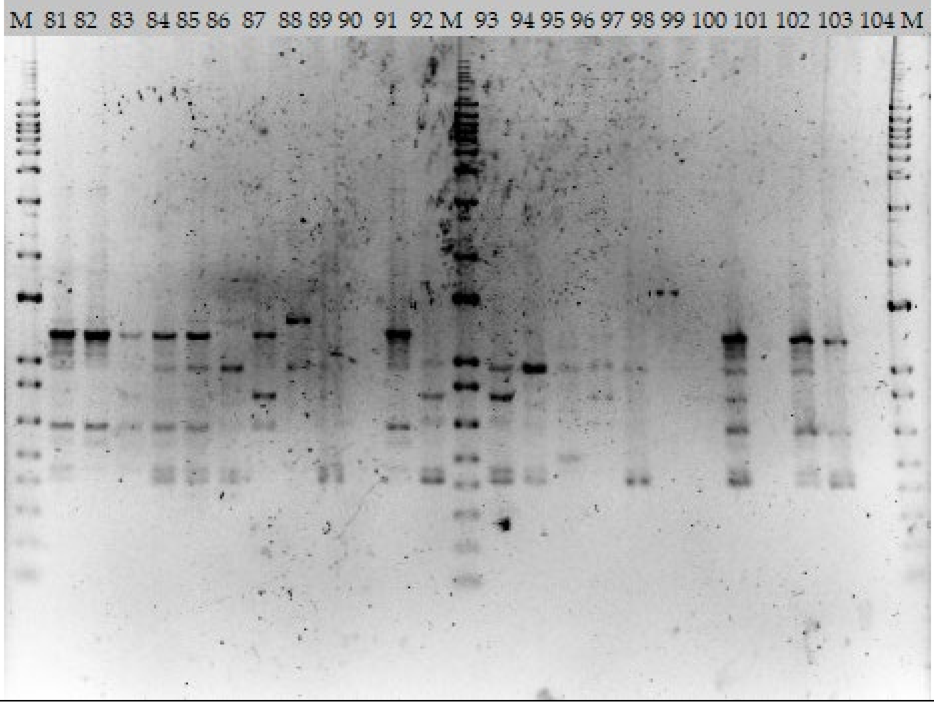
M 81 82 83 84 85 86 87 88 89 90 91 92 M 93 94 95 96 97 98 99 100 101 102 103 104 M

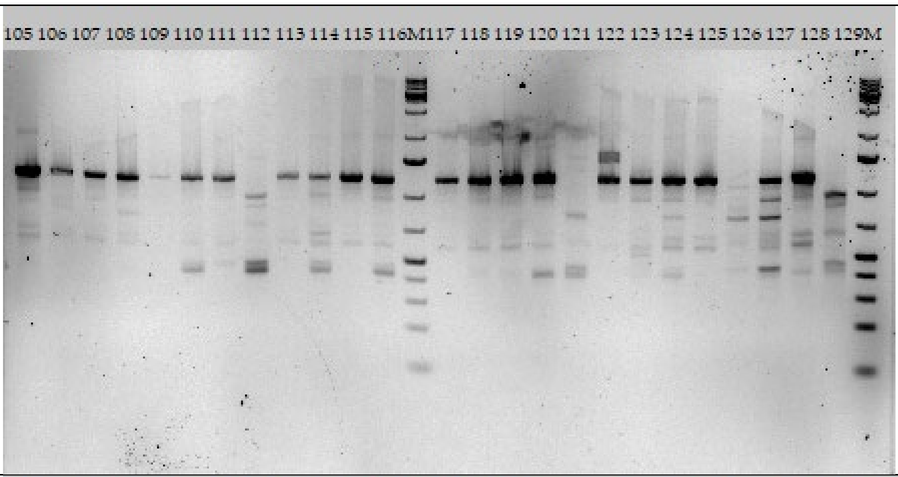
105 106 107 108 109 110 111 112 113 114 115 116M117 118 119 120 121 122 123 124 125 126 127 128 129M

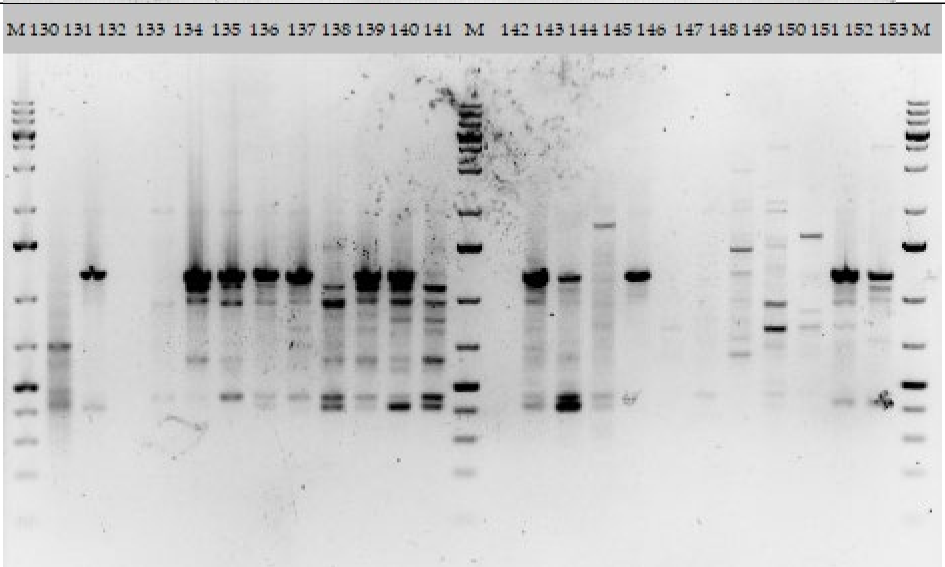
M 130 131 132 133 134 135 136 137 138 139 140 141 M 142 143 144 145 146 147 148 149 150 151 152 153 M

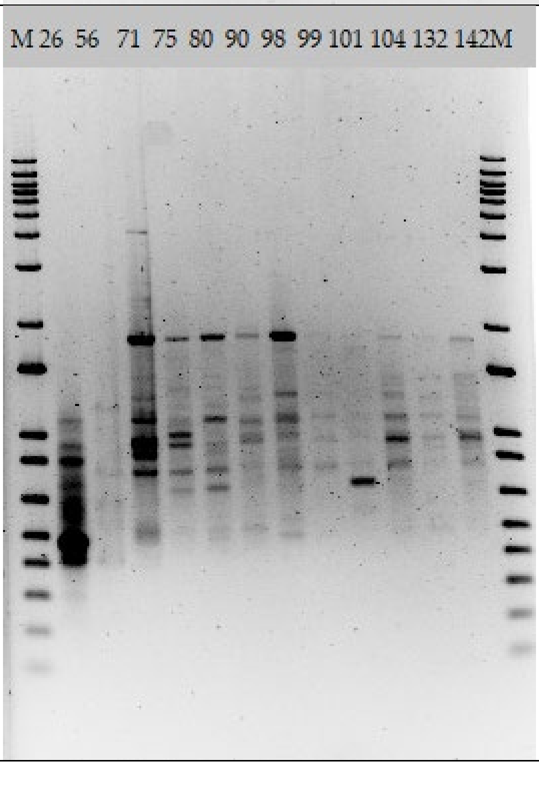
M 26 56 71 75 80 90 98 99 101 104 132 142M

OPN-09	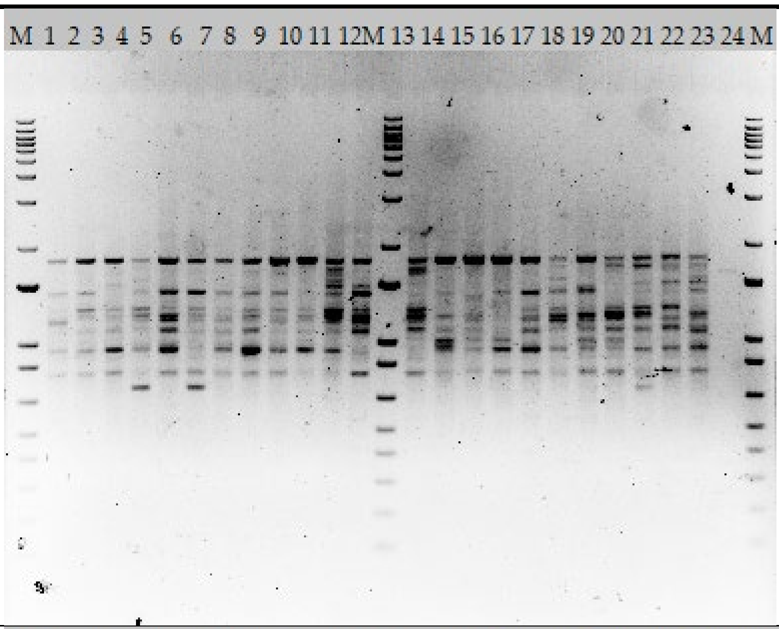 M 1 2 3 4 5 6 7 8 9 10 11 12M 13 14 15 16 17 18 19 20 21 22 23 24 M
	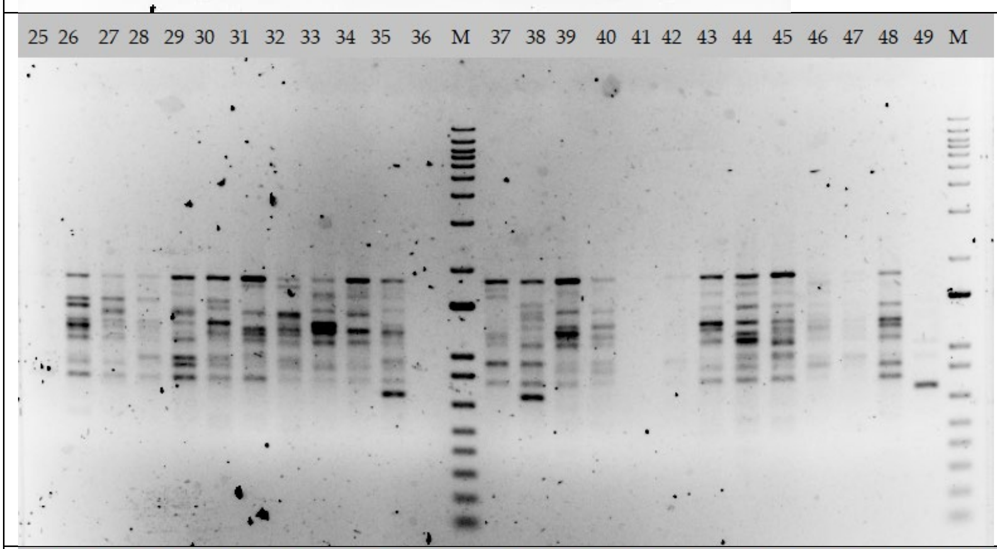 25 26 27 28 29 30 31 32 33 34 35 36 M 37 38 39 40 41 42 43 44 45 46 47 48 49 M
	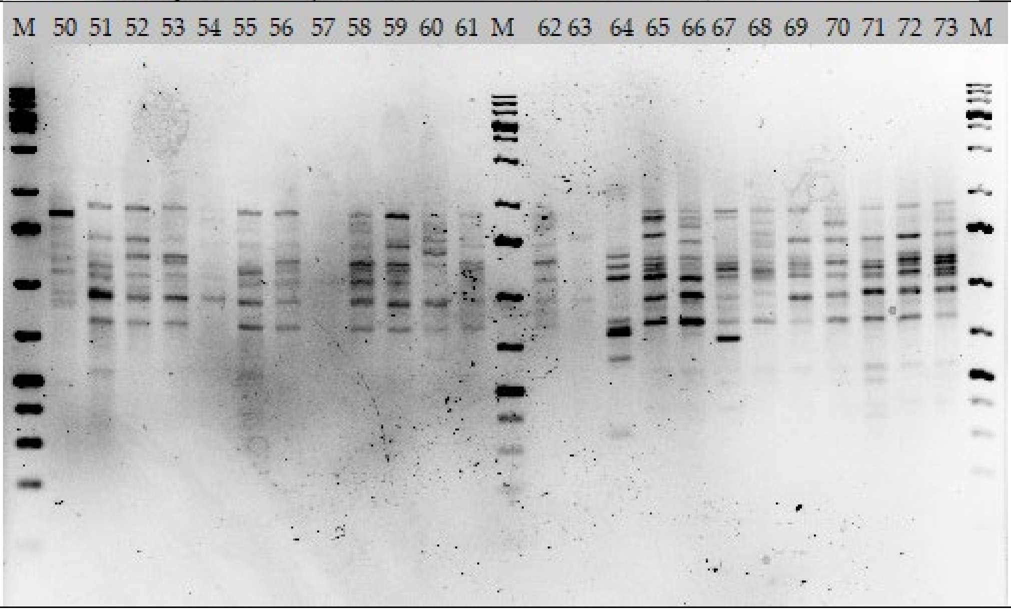 M 50 51 52 53 54 55 56 57 58 59 60 61 M 62 63 64 65 66 67 68 69 70 71 72 73 M

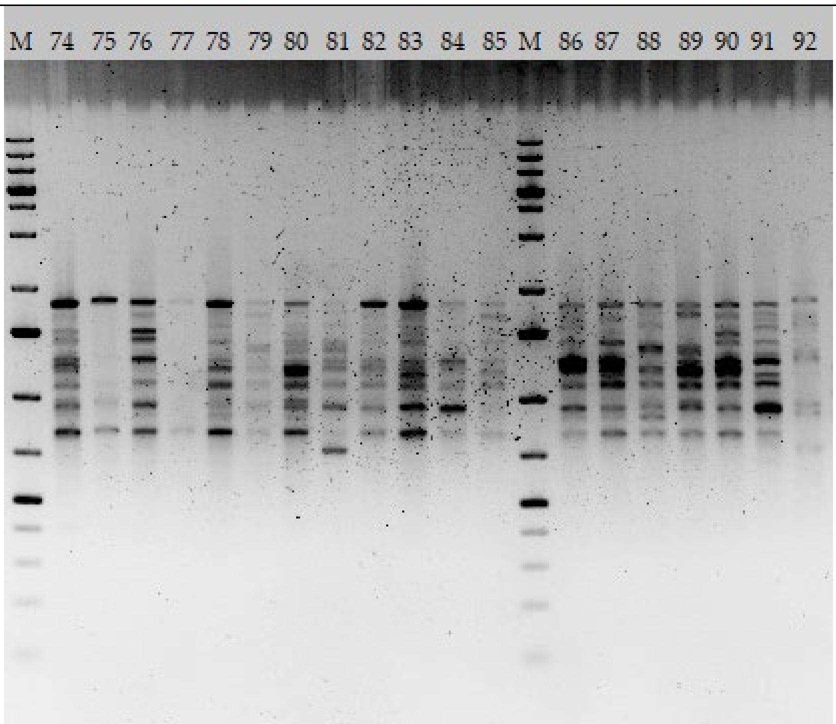
M 74 75 76 77 78 79 80 81 82 83 84 85 M 86 87 88 89 90 91 92

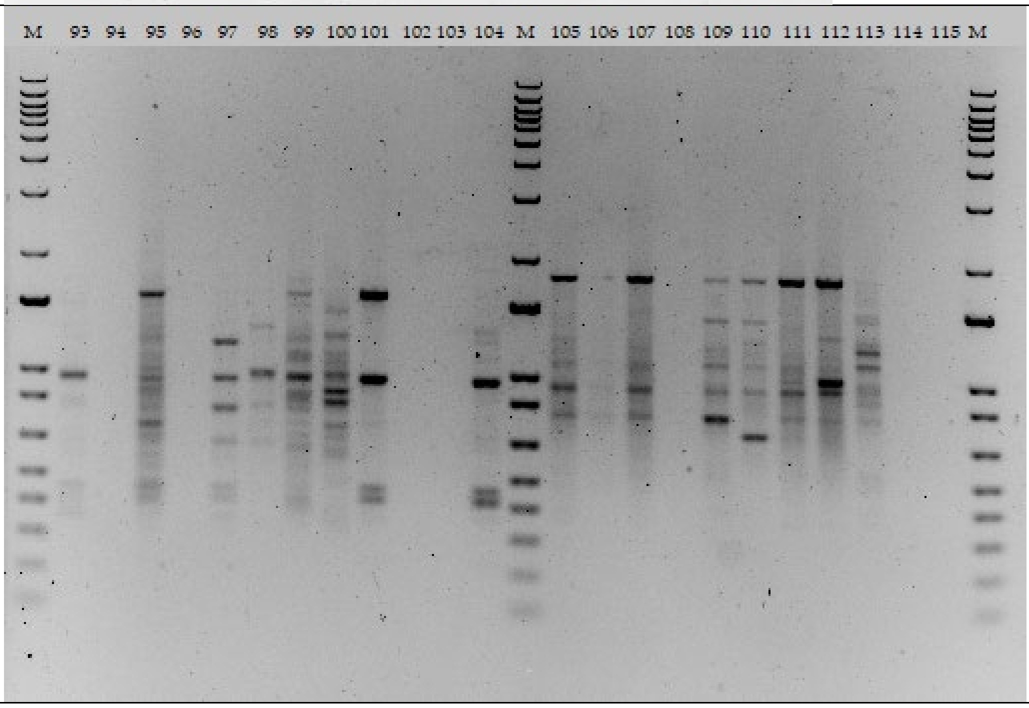
M 93 94 95 96 97 98 99 100 101 102 103 104 M 105 106 107 108 109 110 111 112 113 114 115 M

	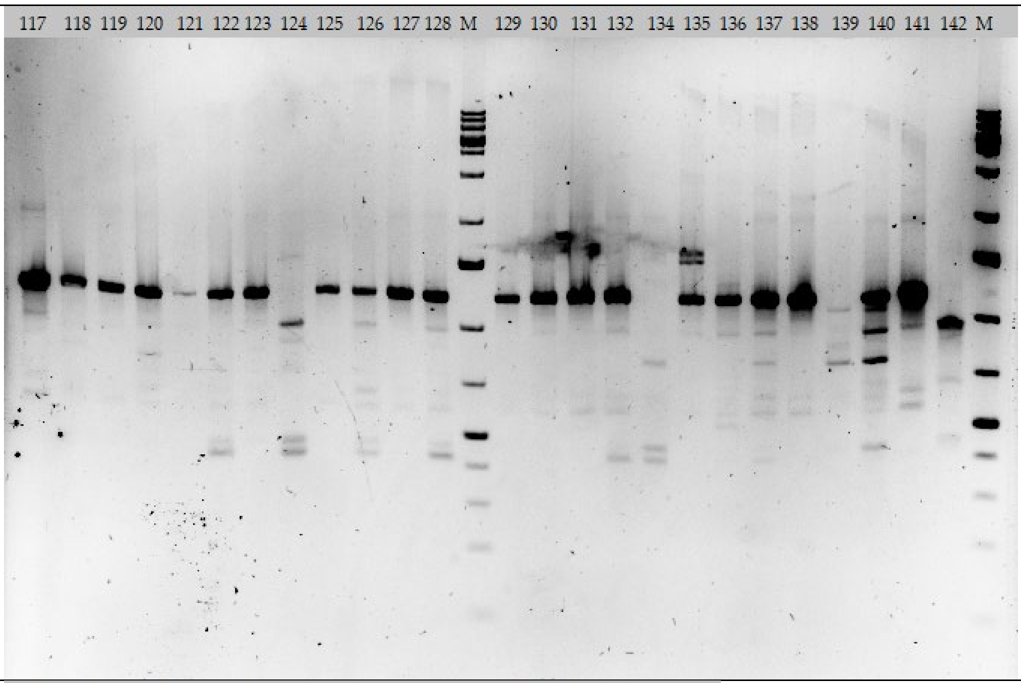
	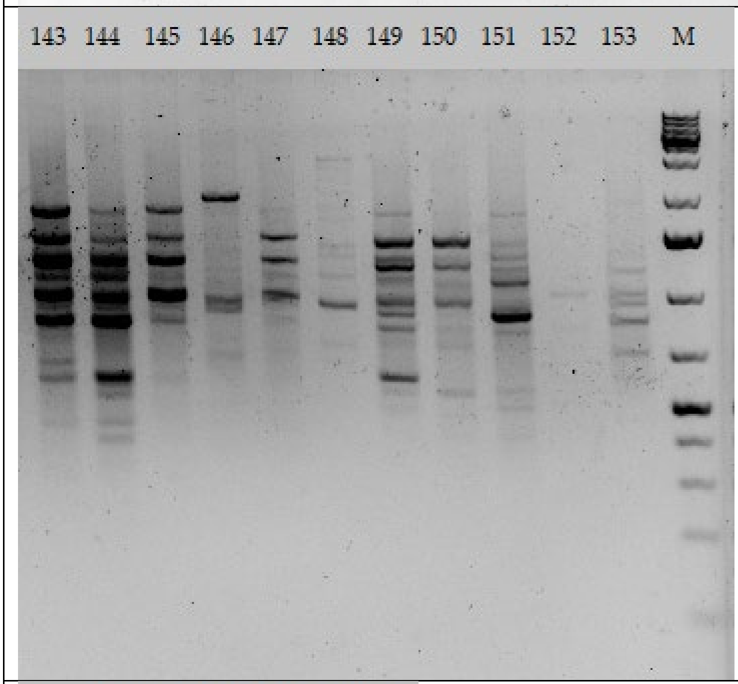
	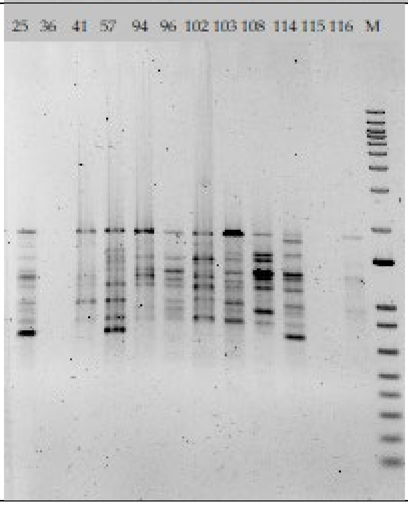 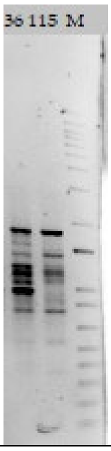

117 118 119 120 121 122 123 124 125 126 127 128 M 129 130 131 132 134 135 136 137 138 139 140 141 142 M
143 144 145 146 147 148 149 150 151 152 153 M
25 36 41 57 94 96 102 103 108 114 115 116 M
36 115 M

OPY-15	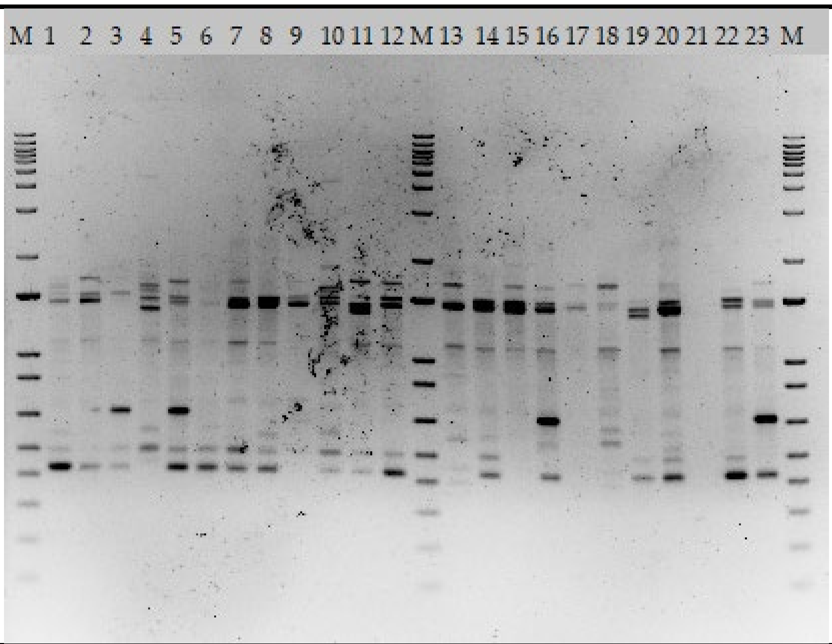 M 1 2 3 4 5 6 7 8 9 10 11 12 M 13 14 15 16 17 18 19 20 21 22 23 M
	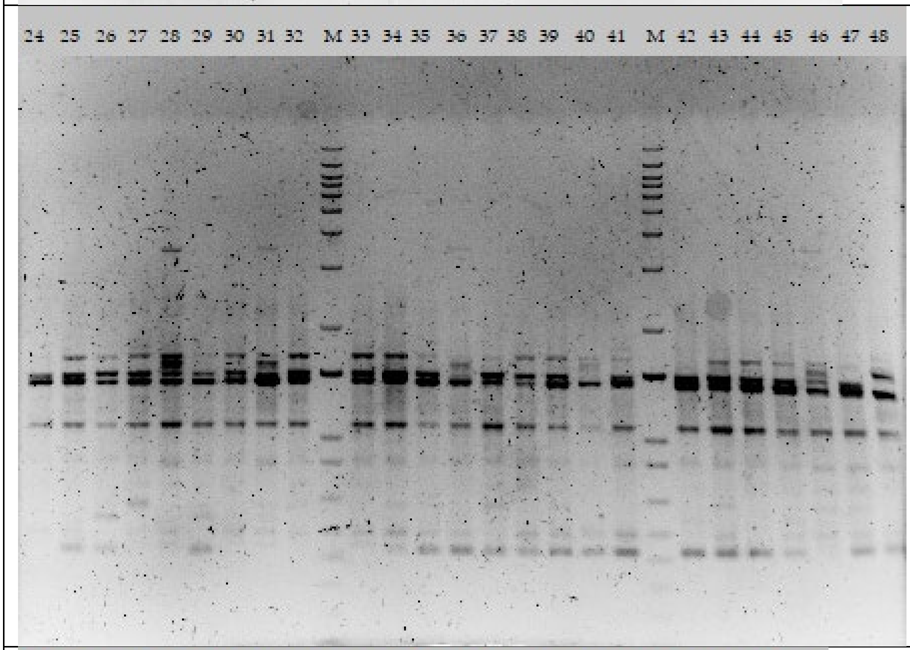 24 25 26 27 28 29 30 31 32 M 33 34 35 36 37 38 39 40 41 M 42 43 44 45 46 47 48
	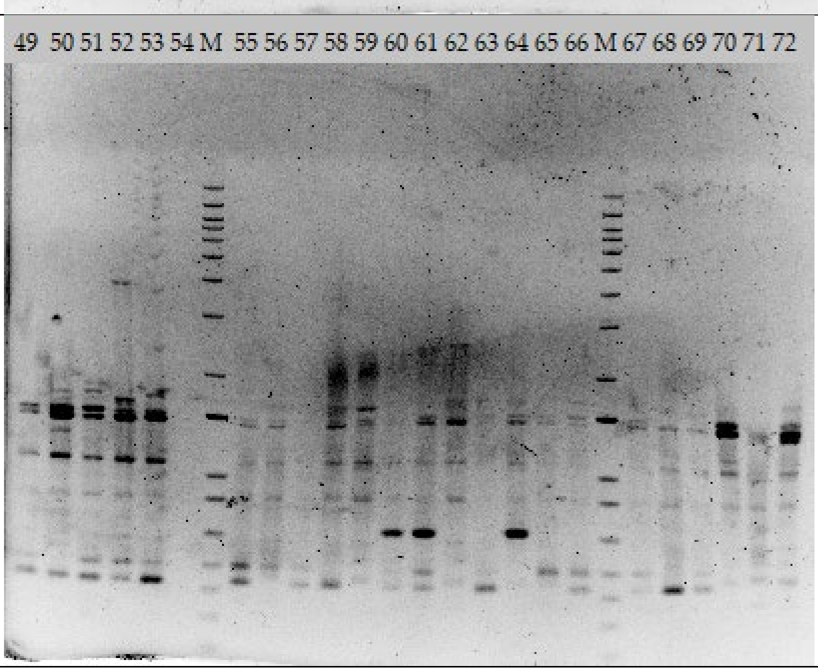 49 50 51 52 53 54 M 55 56 57 58 59 60 61 62 63 64 65 66 M 67 68 69 70 71 72

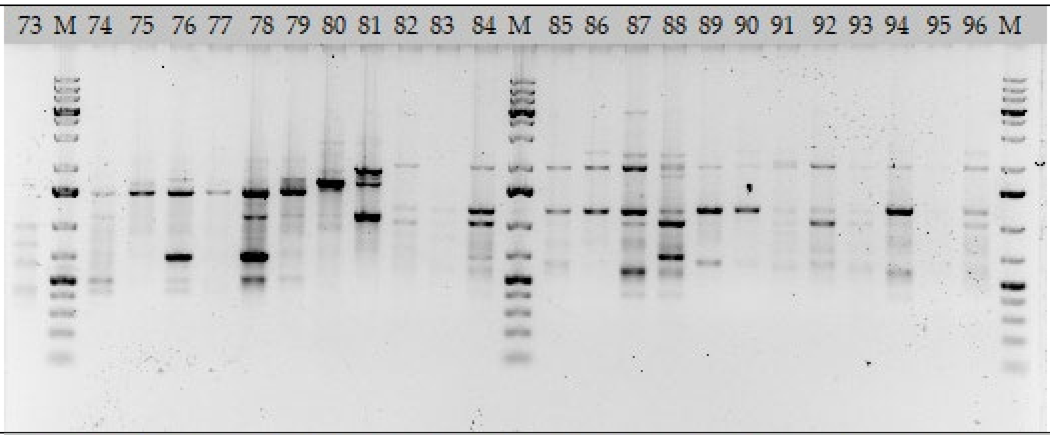
73 M 74 75 76 77 78 79 80 81 82 83 84 M 85 86 87 88 89 90 91 92 93 94 95 96 M

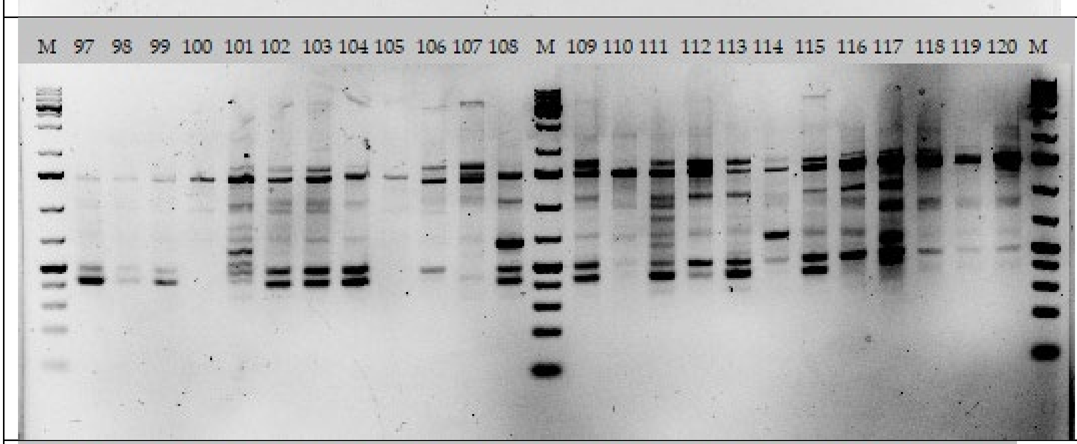
M 97 98 99 100 101 102 103 104 105 106 107 108 M 109 110 111 112 113 114 115 116 117 118 119 120 M

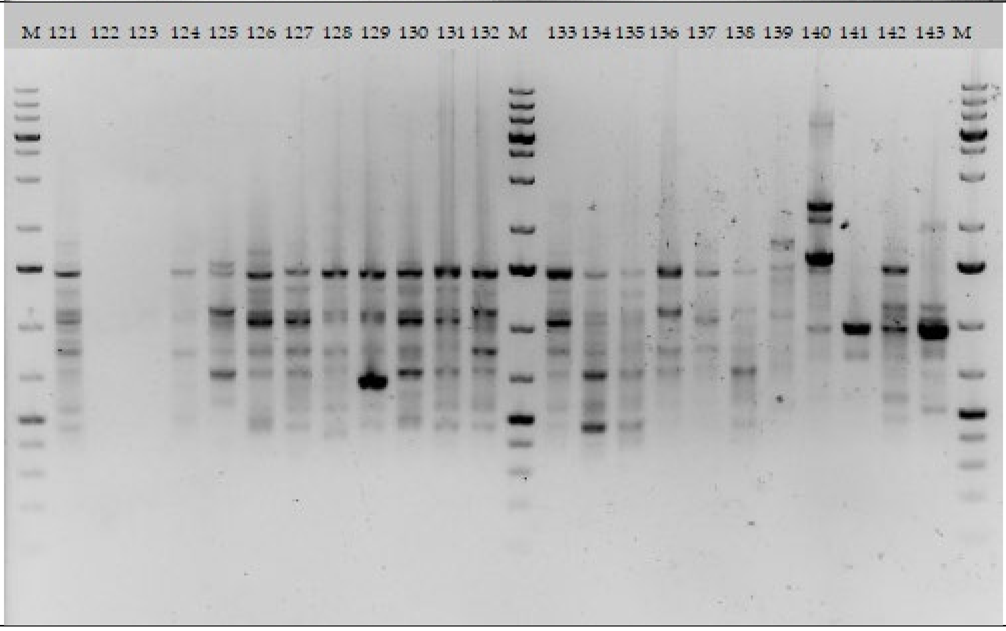
M 121 122 123 124 125 126 127 128 129 130 131 132 M 133 134 135 136 137 138 139 140 141 142 143 M

	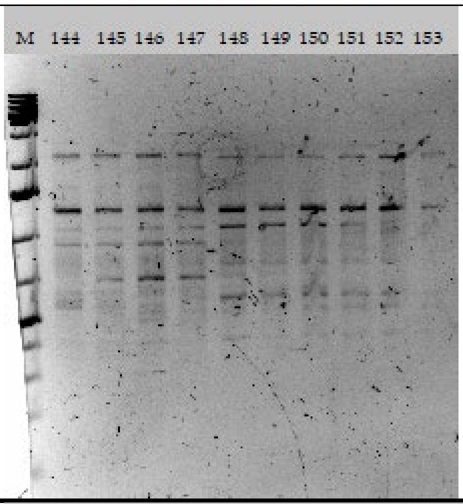 M 144 145 146 147 148 149 150 151 152 153 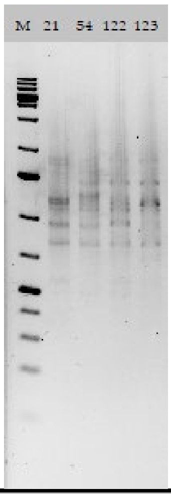 M 21 54 122 123
OPAN-03	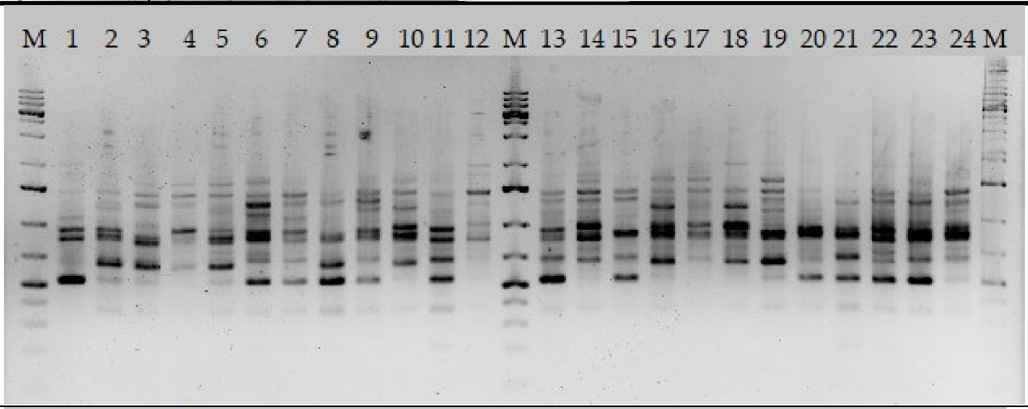 M 1 2 3 4 5 6 7 8 9 10 11 12 M 13 14 15 16 17 18 19 20 21 22 23 24 M
	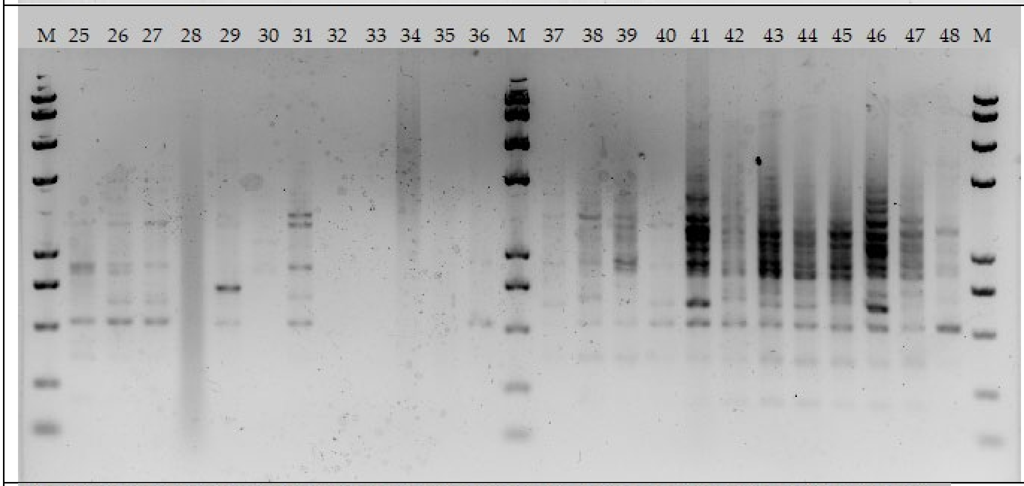 M 25 26 27 28 29 30 31 32 33 34 35 36 M 37 38 39 40 41 42 43 44 45 46 47 48 M
	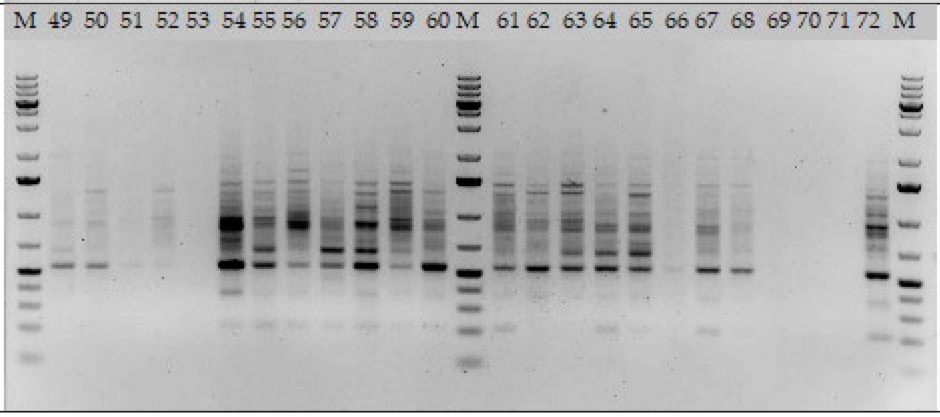 M 49 50 51 52 53 54 55 56 57 58 59 60 M 61 62 63 64 65 66 67 68 69 70 71 72 M

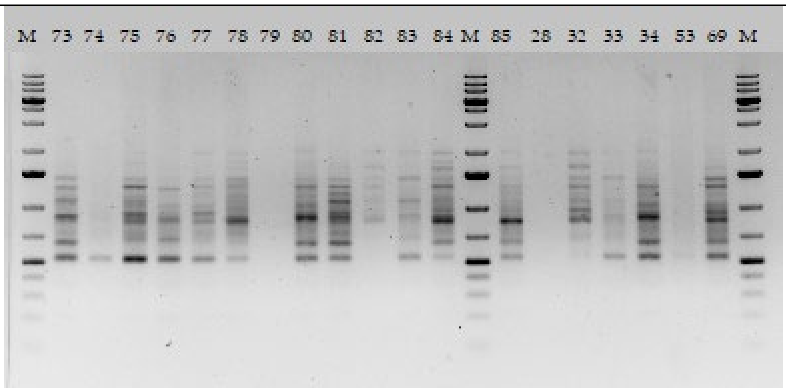
M 73 74 75 76 77 78 79 80 81 82 83 84 M 85 28 32 33 34 53 69 M

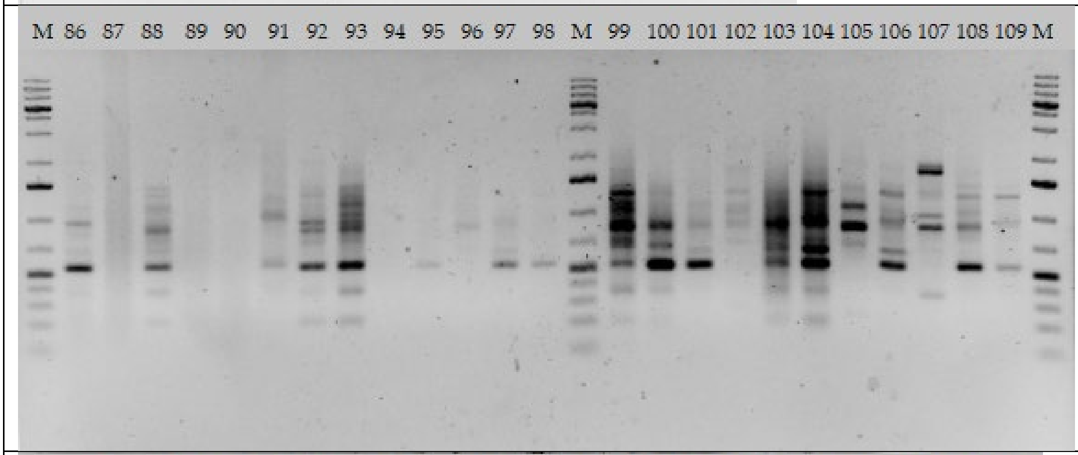
M 86 87 88 89 90 91 92 93 94 95 96 97 98 M 99 100 101 102 103 104 105 106 107 108 109 M

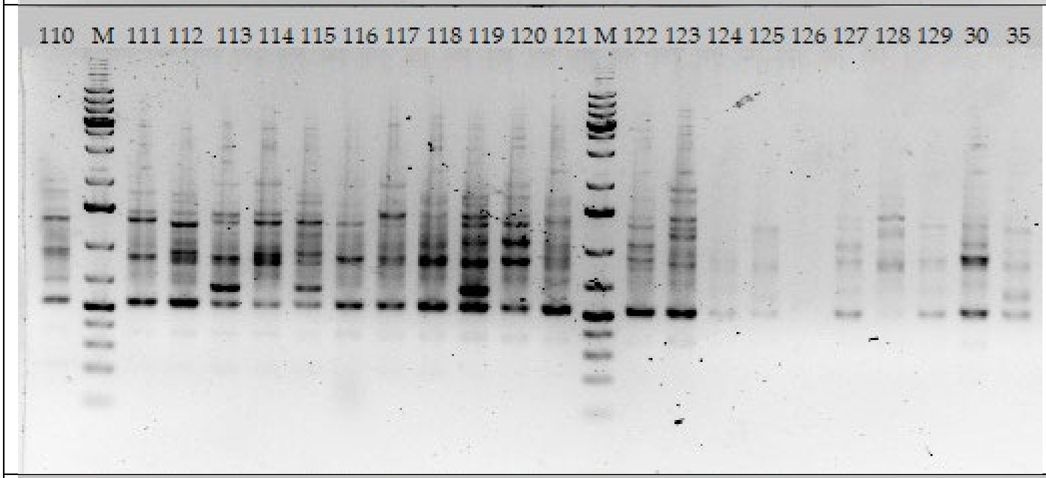
110 M 111 112 113 114 115 116 117 118 119 120 121 M 122 123 124 125 126 127 128 129 30 35

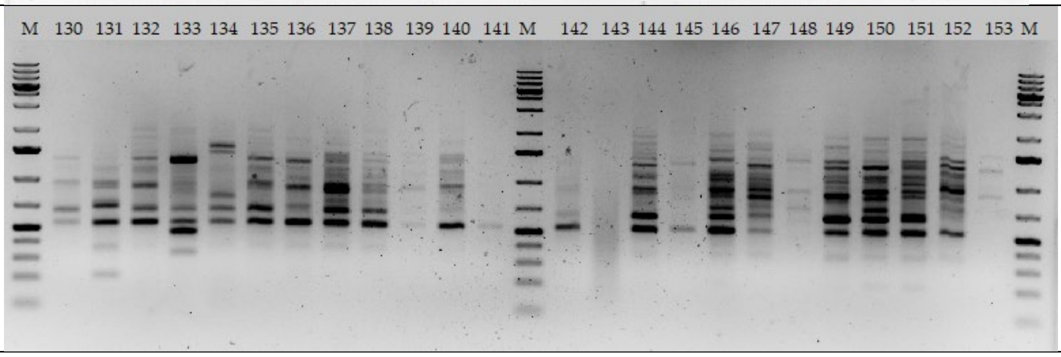
M 130 131 132 133 134 135 136 137 138 139 140 141 M 142 143 144 145 146 147 148 149 150 151 152 153 M

	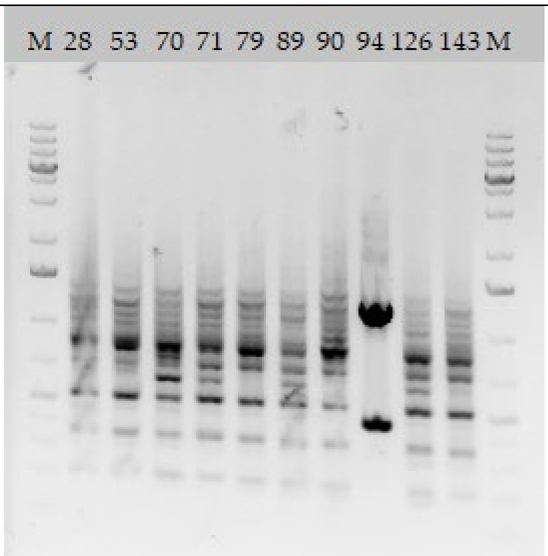M 28 53 70 71 79 89 90 94 126 143 M
UBC378	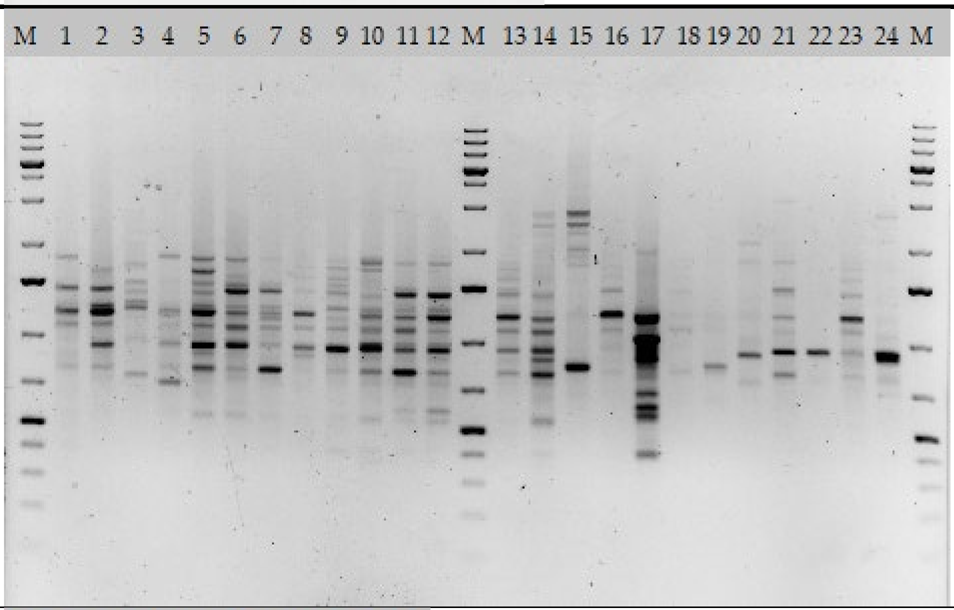M 1 2 3 4 5 6 7 8 9 10 11 12 M 13 14 15 16 17 18 19 20 21 22 23 24 M
	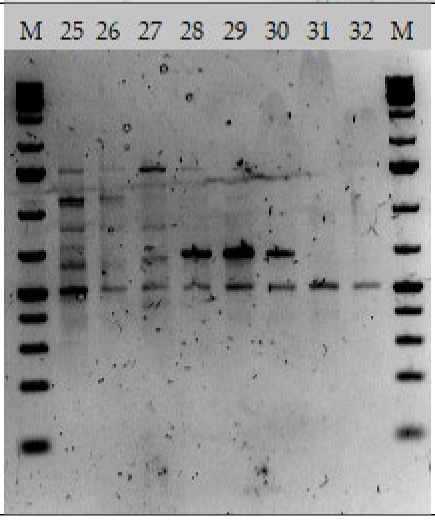M 25 26 27 28 29 30 31 32 M

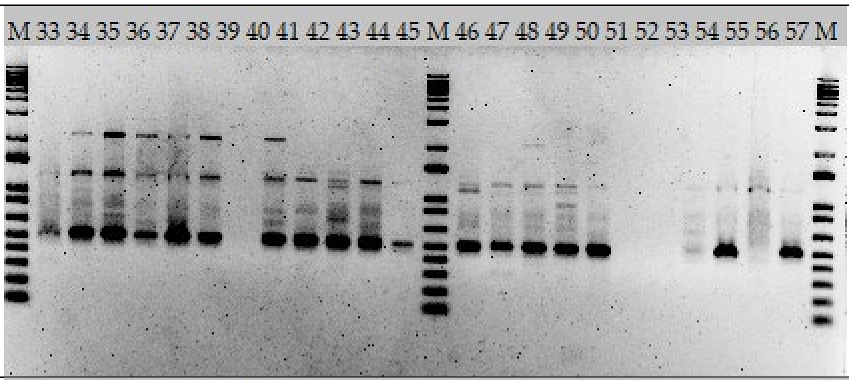
M 33 34 35 36 37 38 39 40 41 42 43 44 45 M 46 47 48 49 50 51 52 53 54 55 56 57 M

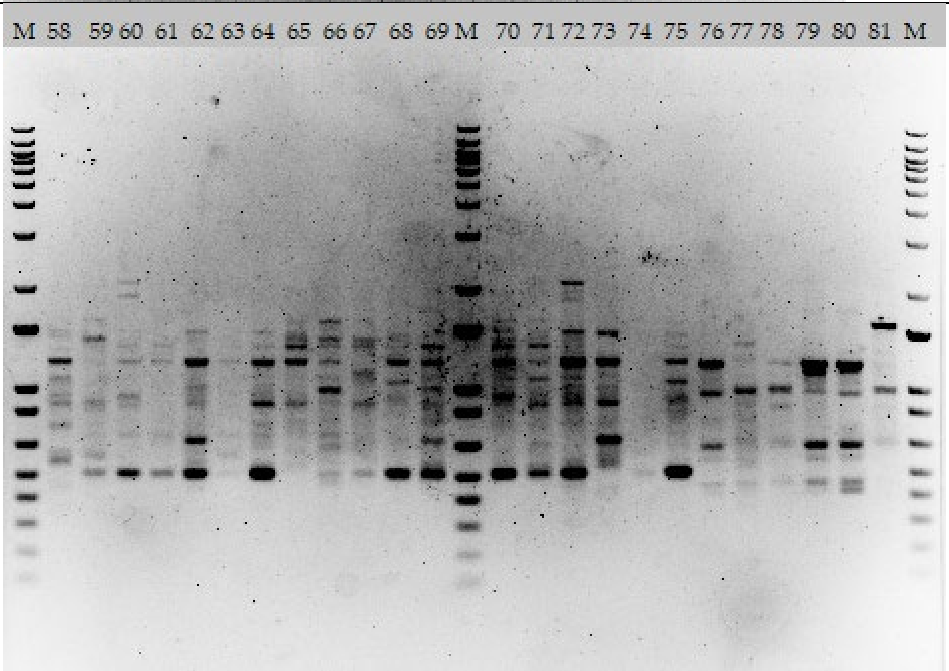
M 58 59 60 61 62 63 64 65 66 67 68 69 M 70 71 72 73 74 75 76 77 78 79 80 81 M

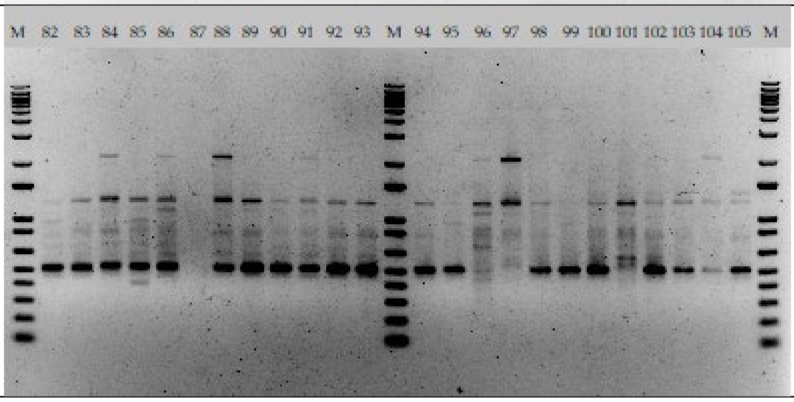
M 82 83 84 85 86 87 88 89 90 91 92 93 M 94 95 96 97 98 99 100 101 102 103 104 105 M

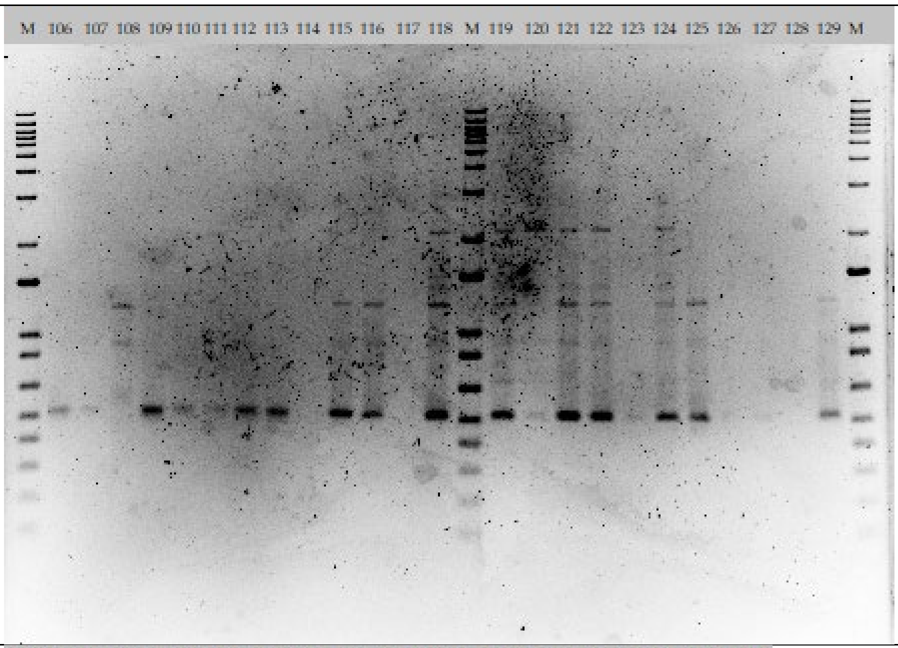
M 106 107 108 109 110 111 112 113 114 115 116 117 118 M 119 120 121 122 123 124 125 126 127 128 129 M

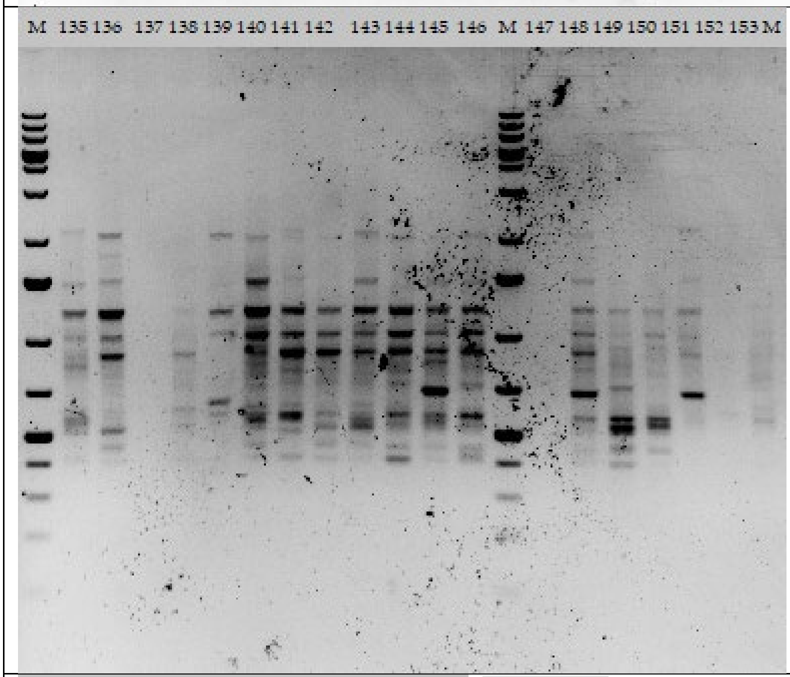
M 135 136 137 138 139 140 141 142 143 144 145 146 M 147 148 149 150 151 152 153 M

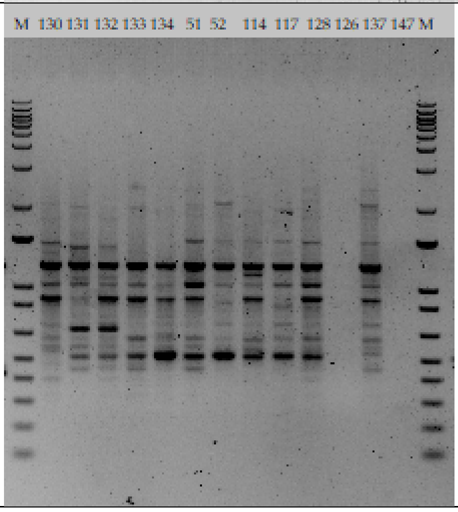
M 130 131 132 133 134 51 52 114 117 128 126 137 147 M

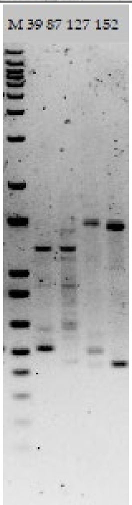
M 39 87 127 152
